# Feasible intervention combinations for achieving a safe exit of the Zero-COVID policy in China and its determinants: an individual-based model study

**DOI:** 10.1186/s12879-023-08382-x

**Published:** 2023-06-12

**Authors:** Qu Cheng, Xingjie Hao, Degang Wu, Qi Wang, Robert C. Spear, Sheng Wei

**Affiliations:** 1 Department of Epidemiology and Biostatistics, School of Public Health, Tongji Medical College, Huazhong University of Science and Technology, Wuhan, Hubei China; 2 Departments of Epidemiology and Biostatistics, School of Public Health, Guangzhou Medical University, Guangzhou, China; 3 Division of Environmental Health, of Public Health, University of California, Berkeley, CA USA

**Keywords:** China, SARS-CoV-2, Zero-COVID strategy, Interventions, Safe exit

## Abstract

**Background:**

Although several pathways have been proposed as the prerequisite for a safe phase-out in China, it is not clear which of them are the most important for keeping the mortality rate low, what thresholds should be achieved for these most important interventions, and how the thresholds change with the assumed key epidemiological parameters and population characteristics.

**Methods:**

We developed an individual-based model (IBM) to simulate the transmission of the Omicron variant in the synthetic population, accounting for the age-dependent probabilities of severe clinical outcomes, waning vaccine-induced immunity, increased mortality rates when hospitals are overburdened, and reduced transmission when self-isolated at home after testing positive. We applied machine learning algorithms on the simulation outputs to examine the importance of each intervention parameter and the feasible intervention parameter combinations for safe exits, which is defined as having mortality rates lower than that of influenza in China (14.3 per 100, 000 persons).

**Results:**

We identified vaccine coverage in those above 70 years old, number of ICU beds per capita, and the availability of antiviral treatment as the most important interventions for safe exits across all studied locations, although the thresholds required for safe exits vary remarkably with the assumed vaccine effectiveness, as well as the age structure, age-specific vaccine coverage, community healthcare capacity of the studied locations.

**Conclusions:**

The analytical framework developed here can provide the basis for further policy decisions that incorporate considerations about economic costs and societal impacts. Achieving safe exits from the Zero-COVID policy is possible, but challenging for China’s cities. When planning for safe exits, local realities such as the age structure and current age-specific vaccine coverage must be taken into consideration.

**Supplementary Information:**

The online version contains supplementary material available at 10.1186/s12879-023-08382-x.

## Background

Since the beginning of the COVID-19 pandemic, China implemented the Zero-COVID policy which seeks to completely stop community transmission by very stringent interventions like lockdowns, mobility restrictions, mass testing, contact tracing, case isolation, close contact quarantine, and border quarantine [1, 2]. Although this policy successfully enabled the nation to keep the incidence and mortality rate of COVID-19 at very low levels, the emergence of the Omicron variant brought new challenges and opportunities. On one hand, the extremely high transmissibility of Omicron made the complete control of community transmission almost impossible and the Zero-COVID policy unsustainable. On the other hand, the remarkably reduced risk of severe clinical outcomes among patients infected by the Omicron variant, especially among the vaccinated, suggests that it was no longer necessary to prevent cases through stringent interventions [3]. Under these circumstances, China started to loosen the Zero-COVID policy gradually in December, 2022.

Several public health measures were considered as the possible way out of the Zero-COVID policy, including increasing vaccine coverage in the oldest age groups, stockpiling antivirals, and increasing the healthcare capacities temporarily for the infected [4]. However, it is still not clear which of these options is the most effective and to what extent healthcare resources should be prepared in order to maintain an acceptably low mortality rate of COVID-19.

A few studies have modelled the transmission of the Omicron variant in China [5–7], but they examined the resulting incidence and mortality rates of only limited combinations of interventions, instead of addressing a comprehensive framework for identifying all possible intervention combinations as the basis for further policy decisions that incorporating considerations about economic costs and societal impacts. Furthermore, these studies did not take into account that only cases being detected by the healthcare or disease surveillance systems, instead of all cases, can be treated. They usually neglected the excessive mortality rates induced by overburdened hospitals as well.

Here, we develop an individual-based model and an analytical framework to explore if it is possible, and what would make it possible, to safely exit the Zero-COVID policy after considering the population age composition and vaccine coverage as well as the community healthcare capacity. We defined a safe exit as keeping the mortality rate lower than that of influenza in China (14.3 per 100,000 persons) [8], a common respiratory illness with which COVID-19 is usually compared. We chose to compare the mortality rate instead of the infection fatality rate (IFR), since mortality rate reflects not only the virulence, but also the transmissibility of the pathogen. The mortality rate can still be considerable, when the transmissibility is high and a large proportion of the population is infected, even if the IFR is low. Our model is able to account for waning vaccine-induced immunity, increased mortality rates when hospitals are overburdened, and reduced transmission when self-isolated at home after testing positive. We first examined the results for China as a whole, then examined three representative cities Shanghai, Shenzhen, and Shiyan which differ in healthcare capacity (Table S1), age structure (Fig. S1), and age-specific vaccine coverage (Fig. S2).

## Methods

### Synthetic population for China and the three representative cities

We first generated a synthetic population of 500,000 individuals to represent the age composition and vaccine coverage at each location. Sensitivity analyses with population sizes of 1, 2, and 5 million showed that the median mortality rate is insensitive to the size of the synthetic population (Fig. S3). We considered a total of 16 age groups (0–4, 5–9, …, 70–74, and above 75 years old) and 4 vaccine dose groups (0–3 doses). For each location, we first assigned individuals to an age group with probabilities proportionate to the share of each age group in the general population of that location in 2020 (Fig. S1). We then assigned each individual a vaccine dose group according to probabilities proportionate to the dose-specific vaccine coverage in that age group for that location (Fig. S2). Besides age composition and vaccine coverage, we also assumed that the availability of healthcare resources varied with location (Table S1).

We assumed that the social contact pattern only depends on age but not vaccination status. We obtained the raw social contact matrices between age groups for *all* and only *home* settings from a survey conducted in Shanghai in 2017 before the pandemic and smoothed them with 8-dimension tensor-product spline bases as in previous literature [9, 10]. Fig. S4 shows the smoothed contact matrices for *all* and only *home* settings. We assumed that the contact patterns after the end of Zero-COVID policy returned to the level before the pandemic. We further assumed that once identified, the cases would self-isolate at home and have less contacts with their family members, so their contact rates are only 20% of the average contact rate in *home* setting [11]. We also ran a sensitivity analysis in which we assumed no reduction in the home contact rate after detection (*No self-isolation* scenario in Table S2).

### Model description

A discrete-time individual-based model was developed to simulate the transmission dynamics of the Omicron variant in the synthetic population and the disease progression of each case, given their age group, vaccination status, antiviral treatment usage, and place of recovery (home, hospitals, or ICU). All individuals were considered to be susceptible at the beginning of the simulation, given the low cumulative prevalence before December, 2022 in China [12, 13].

**Fig. 1:**
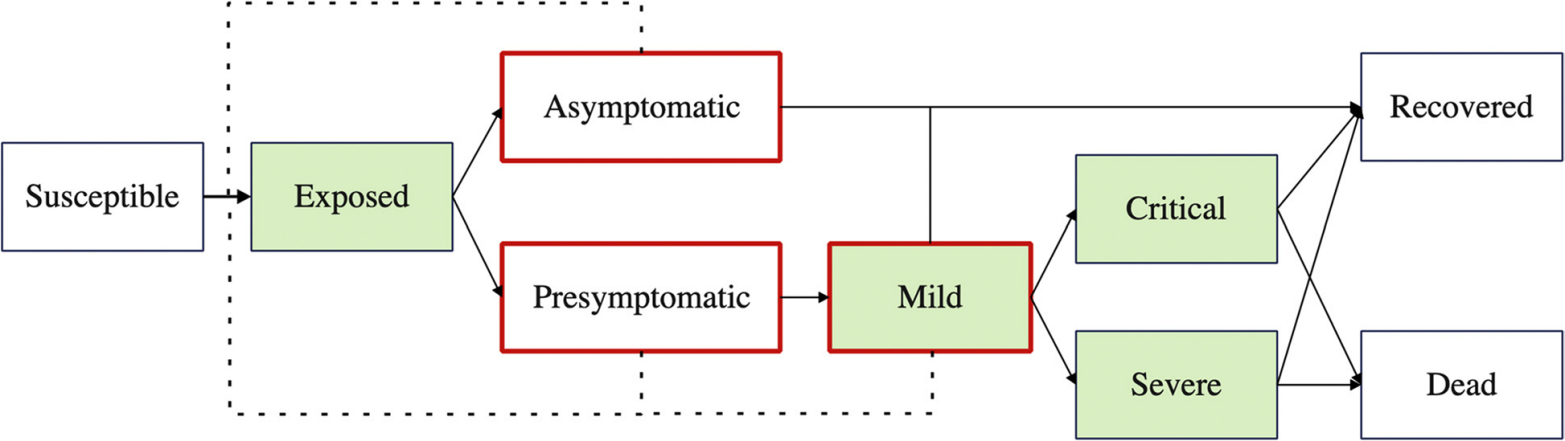
Schematic of the individual-based model. The probabilities of proceeding to each compartment downstream of the green compartments depends on the individual’s age group and vaccination status (Tables S3, S4 and ST1.2). The proportions of severe and critical cases that die also depend on their places of recovery (see details in *Interventions*). We assumed only compartments with red borders are transmissible

Individuals were partitioned into nine epidemiological states (Fig. 1). We assumed that hospital beds were needed to care for severe cases, while ICU beds were needed for critical cases. Susceptibles can be infected through contacts with asymptomatic, presymptomatic, and mild cases, but not severe and critical cases, since they are highly likely to be identified and isolated due to their obvious symptoms. Asymptomatic and presymptomatic cases can be identified through screenings before hospital admissions for non-COVID conditions, while symptomatic cases can be identified through at-home rapid antigen testing. We assumed a detection rate of 1.5 percent for asymptomatic and presymptomatic cases [14] and 75 percent for symptomatic cases by assuming a full coverage of rapid antigen tests with a sensitivity of 0.75 [15]. We further used a 75 percent coverage in a sensitivity analysis (*75% testing* scenario Table S2).

We assumed that 0.6 cases were imported to the synthetic population each day, according to the per capita importation rate to Hubei in early December, 2022 (Dr. Yeqing Tong, personal communication, 2022 Dec 5). We simulated the transmission dynamics for a whole year (365 days) after the first importation at a time step of 4 h (1/6 day). At each time step, we first estimated the force of infection ${\lambda }_{i, t}$ for each susceptible *i* at time *t,* which represents the rate at which *i* moves from the susceptible to the exposed group. The force of infection ${\lambda }_{i, t}$ depends on mask coverage, social contact pattern, vaccine coverage and waning of vaccine-induced immunity of the community, as well as the transmission probability per contact of the pathogen, ${\beta }_{0}$. We calibrated ${\beta }_{0}$ to have a R_0_ of 7 for the Omicron variant according to previous studies [7, 16]. We further set R_0_ to 5 and 10 in the sensitivity analysis (*R*_*0*_ = *5* and* R*_*0*_ = *10* scenario Table S2). The details about estimating ${\lambda }_{i, t}$ are presented in S1 Text. We then determined if this individual was infected in this time step through sampling from a Bernoulli distribution with a success probability of ${\lambda }_{i, t}$.

The disease progression of each individual (e.g., from Exposed to Asymptomatic or Presymptomatic, from Mild to Severe, Critical or Recovered) was sampled from categorical distributions with transition probabilities depending on age group, vaccination dose, drug intake and place of recovery. Younger age groups (Table S3) and individuals with more vaccine doses (Table S4) were assumed to have a lower probability of proceeding to symptomatic, severe, critical, and deceased cases according to a previous study [5]. Taking antiviral treatments or being cared by healthcare teams can further reduce the probability of proceeding to severe clinical outcomes (See *Interventions* for details). The duration of time spent in each state for each individual was sampled from the Gamma distribution (Table S5) and was assumed to be independent of age and vaccination status. To account for stochasticity, 48 repetitions were run for each simulation. The total number of deaths at the end of each repetition was recorded to estimate the median mortality rate.

To test the validity of our model, we compared the simulated age-specific IFRs from the model based on Shanghai’s age structure and vaccine coverage, with the values observed in Shanghai during an Omicron outbreak in the spring of 2022 [17]. We assumed a mask coverage of 75 percent according to a survey [18], and that no hospital strains occurred during the period.

### Interventions

We examined the impacts of four types of interventions with relatively low societal disturbance that could have been implemented after the end of the Zero-COVID policy, including increasing facial covering, increased healthcare capacity (including hospital and ICU beds), vaccine coverage, and antiviral treatment coverage. We did not include lockdowns, school and workplace closures, travel restrictions, mass testing, mandatory case isolation, and close contact quarantine after contact tracing, because they are less favorable by both the policy makers and the general public. The implementation of these interventions in the model are summarized below.

#### Facial covering

Facial covering was required for entering public places in China before December, 2022 [19]. We assumed this is retained after the end of the Zero-COVID policy due to its low economic and societal impacts. According to a recent study in community settings, a mask coverage of *p*_*m*_ can reduce the transmission by 1-0.75^*Pm*^ [20]. Therefore, we scaled down the force of infection to 0.75^*Pm*^ of its original value in the simulation (see the first term in the equation for force of infection in S1 Text). Here, the range of the mask coverage (the parameter *Mask*) is set to be 0 to 1, representing that 0 to 100 percent of the population wear masks.

#### Healthcare capacity

Once identified, we assumed that severe and critical cases are admitted to hospital and to ICU beds, respectively. If no hospital beds are available for severe cases, they remain at home, but their mortality rate will increase to 10 times the mortality rate of those hospitalized. We used mortality rates of 2 and 5 times in the sensitivity analysis (2*Hosp. Mort. and 5*Hosp. Mort. scenarios in Table S2). If no ICU beds are available for critical cases, they are always assumed to die, since their mortality rate is already as high as 30 to 40 percent even when having the needed ICU care [21–23]. For each location, the numbers of ICU and hospital beds per capita (the parameters *ICU* and *Hospital*) are assumed to range between the current values at that location (Table S1) to the worldwide maximum (48 ICU beds per 100,000 [24] and 14.4 hospital beds per 1000 [25]).

#### Vaccination

Inactivated vaccines are used in China. Although their ability in preventing infections caused by the Omicron variant is limited, their effectiveness in preventing severe clinical outcomes is high [26]. The first, second, and booster doses reduce the susceptibility to infection by only 3.1, 7.0, and 13.1 percent, respectively (S1 Text); while they reduce the overall probability of death by 54.6, 74.8 and 88.8 percent, respectively (Tables S3) [5]. The vaccine-induced immunity is assumed to wane across time at a constant rate until a stable rate is reached (S1 Text) [27]. We assumed that the first and second doses have almost no impact on the probability of onward transmission from a recipient, while the booster can reduce it by 5.3 percent (S1 Text) [5]. To examine the effect of increasing vaccination coverage in different age groups on the probability of safe exit, we varied four parameters, *ΔVac. 0–19*, *ΔVac. 20–59*, *ΔVac. 60–69*, and *ΔVac. 70above*, representing the proportion of individuals having one additional dose in age groups 0–19, 20–59, 60–69, and above 70, between 0 and 1.

#### Antiviral therapy

The neutralizing antibody combination therapy BRII-196/BRII-198 and the combination antiviral therapy nirmatrelvir/ritonavir tablets were recently approved in China although not widely used yet. They can be prescribed to mild cases above 12 years of age and are assumed to be able to reduce hospitalization and death rate by about 80 percent [5]. The coverage of antiviral therapy (the parameter *Antiviral*) is assumed to range between 0 to 1 in the eligible population.

### Possibility of a safe exit

To examine the possibility of keeping the mortality rate of COVID-19 below 14.3 per 100,000 persons (referred to as *safe exit* hereinafter), we first ran the model under the *best-case* intervention scenario, when all parameters were set to their upper bounds, then the *worst-case* scenario, when all parameters were set to their lower bounds (see detailed values in S2 Text). A mortality rate higher than 14.3 per 100,000 persons under the *best-case* scenario suggests that a safe exit is impossible for the location without more stringent interventions like mass testing and contact tracing, while lower than 14.3 per 100,000 persons under the *worst-case* scenario suggests that the current healthcare capacity and vaccine coverage are enough for a safe exit and no further action will be needed.

To assess sensitivity, we repeated the analyses with different coverages of rapid antigen tests, transmission intensities, vaccine effectiveness, relative susceptibility of children to adults, relative infectivity of asymptomatic to symptomatic cases, and increases in mortality rate when not having the required beds (Table S2). Only sensitivity scenarios that end in safe exits under the *best-case* scenario but not the *worst-case* scenario, and having median mortality rates differ significantly from the baseline scenario, are included in further analyses.

### Importance of different interventions in determining the mortality rate

To examine the impact of each intervention on controlling the mortality rate of COVID-19, we first randomly sampled 100 combinations of the 8 intervention parameters using the maximin Latin Hypercube sampling method, which facilitates efficient exploration of the parameter space with a small number of samples [28]. Then for each intervention parameter set, we ran the simulation 48 times to account for stochasticity and estimated the median mortality rate at the end of each simulation. Next, we used the median mortality rate as the dependent variable together with the 100 sets of random samples as the independent variables to fit Gaussian process (GP) models with constant means and quadratic exponential kernels. The GP model assumes that all samples, including the observed and unobserved samples, are jointly multivariate normal, and the covariance between them can be characterized by a predefined function of the distances between them [29]. The prediction for an unobserved sample can be made based on the covariances between pairs of two observed samples, and between pairs of one observed and this unobserved sample. GP models were used here as emulators of the original computationally intensive simulations due to their ability to make predictions with uncertainty in a significantly reduced amount of time [30]. For instance, it takes about 15 min on a cluster with 48 cores of 3.0 GHz Intel Xeon Gold 6248R CPU to obtain the median mortality rate of a specific parameter set through simulations, while only less than one second using a fitted GP model. To ensure that the GP models make accurate predictions, we used a ten-fold cross-validation design. We first divided all data into 10 subsets, each with 10 sets of intervention parameters and their corresponding median mortality rates, then used every combination of 9 of these to train the model and the one remaining as an out-of-bag testing samples to validate the model. After confirming the predictive power of the fitted models on the out-of-sample testing datasets, we again refitted the models with the whole dataset and used them to examine the importance of each intervention parameter through the permutation importance procedure [31]. In that procedure, we first randomly shuffled the values of each intervention parameter one at a time and used them together with the other seven unchanged parameters, to predict the median mortality rate. We then estimated the permutation importance score of the shuffled parameter as increase in the root mean square prediction error when compared with the predictions made by the original parameter values. A larger permutation importance score suggests that the shuffled parameter is more important, since a larger increase in the prediction error suggests that the model relies on this parameter to make accurate predictions.

### Feasible intervention combinations for a safe exit

We tried to identify the feasible intervention combinations for a safe exit through examining the predictions made by GP models on a fine grid of the intervention parameters. However, a major drawback of the GP model is that its predictions reverse quickly to the mean when extrapolating far away from all known data points. Since the mortality rates estimated from the previously sampled 100 sets of intervention parameters rarely reached the desired mortality rate of lower than 14.3 per 100,000 persons (Fig. 4A), we need to obtain more samples from a reduced parameter space that result in mortality rates closer to the desired range. The methods for accomplishing this was described in S2 Text. After obtaining the new samples, we fitted GP models to these new samples, and made predictions on the fine intervention parameter grid to identify the feasible intervention combinations for a safe exit. In order to address the uncertainties inherent in the stochastic nature of the simulation model, we randomly sampled 10 sets of simulated results and fitted a GP model to each set of them. Additionally, to account for prediction uncertainty of the GP models, we generated 100 random samples from the predicted distribution at a new data point using each of these 10 GP models. We then combined these 1000 samples to estimate the 95% credible interval (CI) of the prediction. We provided an interactive interface for the model outputs at https://canalcheng.shinyapps.io/COVID_exit/. To visualize and provide an intuitive understanding of the feasible intervention space, we identified the three most important intervention parameters in the reduced parameter space and fitted simplified Gaussian process models to them with the median mortality rate. Finally, predictions on fine grids of these three parameters were made to visualize the feasible parameter space as heatmaps.

## Results

### Model results

**Fig. 2:**
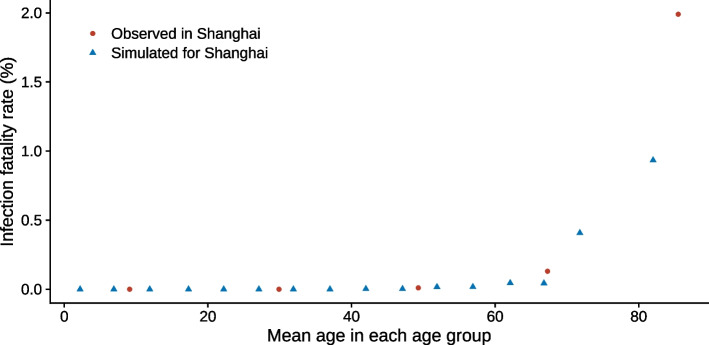
Age-specific infection fatality rates estimated from the simulation (blue triangles) and observed in Shanghai during an Omicron outbreak in the spring of 2022 (red dots) [17]

The overall IFR from the simulation was 0.08% (95% CI: 0.07–0.08%), very close to its observed value of 0.09% (95% CI: 0.09–0.10%). The age-specific IFRs output by the simulation show a similar magnitude and trend as the observed data (Fig. 2) [17]. They were both very low below 60 years old but increased rapidly after that. The large difference in the infection fatality rates of the oldest age group between the simulation and the observation is likely caused by the way the age groups were divided. The oldest age group in our simulation is comprised of individuals over 75 years old, while that in the observed data is those over 80 years old. Given that individuals aged 75–79 years old contributed 39.8% of the population over 75 years old [32] and the IFR increases exponentially with age [33], it is expected that IFR for the oldest age group in our simulation is significantly lower than that of the oldest age group in the observed data.

### Possibility of a safe exit

#### Baseline scenario

Under the best-case intervention scenario (S2 Text), the median mortality rates are 8.0, 9.5, 2,2, and 7.9 per 100,000 persons for China, Shanghai, Shenzhen, and Shiyan, respectively, while under the worst-case intervention scenario (S2 Text), they are 83.7, 150.8, 17.8 and 44.0, respectively. These results suggest that although it is possible for all four locations to have a mortality rate comparable to that of influenza without very stringent interventions, improvements will be needed on the current healthcare capacity and coverages of mask, vaccine and antiviral treatment.

#### Sensitivity analyses

The distribution of the mortality rate is sensitive to only vaccine effectiveness (VE), but not the coverage of rapid antigen test, transmission intensity (R_0_), susceptibility of children, infectivity of asymptomatic case, reduction in home contact rate after detection, and changes in mortality rate when having no access to hospital beds (S3 Text). Therefore, we only kept different VE scenarios in further sensitivity analyses. More specifically, we kept the optimistic VE scenario for all locations and pessimistic VE scenario for only Shenzhen, since safe exits can barely be achieved for the other three locations even under the *best-case* intervention scenario when assuming pessimistic VEs (S3 Text).

### Importance of each intervention in determining the mortality rate

#### Baseline scenario

**Fig. 3:**
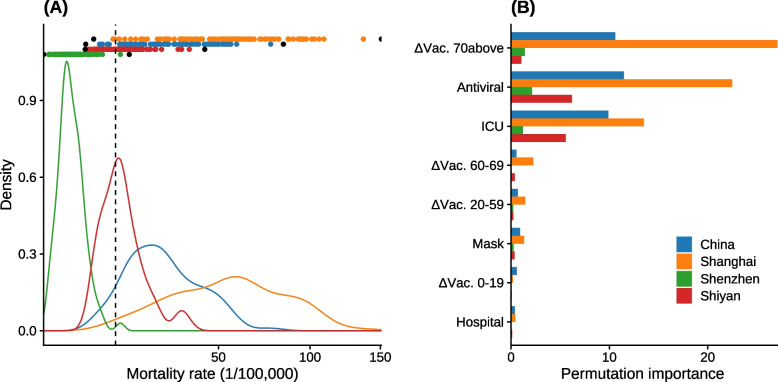
Results of the 100 samples from the full parameter space under the *baseline* scenario. **A** Median mortality rates (colored dots) and their probability density distributions (colored curves) from the simulations. Black dots on the top of the panel show the median mortality rate from the simulations under the best-case (the dot on the left) or the worst-case (the dot on the right) intervention scenario (S2 Text). Vertical dashed line shows the mortality rate of influenza (14.3 per 100,000 persons). **B** Permutation importance of each intervention parameter for predicting the median mortality rate (colored bars). The permutation importance of a parameter represents the amount of increase in the root mean squared prediction error when the randomly shuffled values of this parameter, together with the unchanged values of the other parameters, were used to make the prediction

Under the *baseline* scenario, 7, 2, 99, and 40 out of the 100 random samples from the full parameter space result in safe exits for China, Shanghai, Shenzhen, and Shiyan, respectively; with average median mortality rates of 29.3, 57.2, 5.72, and 16.4 per 100,000 persons (Fig. 3A). Results from the tenfold cross-validation suggest that the fitted Gaussian process emulators have strong and robust predictive power, even on the out-of-bag samples that were not used in training the model (Fig. S5). The GPs fitted to the full datasets were used to examine the importance of each intervention in determining the mortality rate. For all locations, *ΔVac. 70above*, *Antiviral*, and *ICU* are the most important intervention parameters in determining the mortality rate, while *Hospital* and *ΔVac. 0–19* are the least important (Fig. 3B). The permutation importance score of the same intervention parameter for Shanghai is usually greater than that of other locations, since permutation importance measures the amount of increase in the root mean squared prediction error and Shanghai tends to have a larger prediction error due to its higher mortality rate (Fig. 3A).

#### Sensitivity analyses

Results under the *optimistic VE* and *pessimistic VE* scenarios are shown in S3 Text. A majority of the 100 random samples resulted in median mortality rates that are in the desired range for Shenzhen under both the *optimistic VE* and *pessimistic VE* scenarios, and for Shiyan under the *optimistic VE* scenario. Therefore, we did not reduce the parameter space for them, but only the spaces for China and Shanghai. Under all the studied VE scenarios, *ΔVac. 70above*, *Antiviral*, and *ICU* are still the most important parameters.

### Feasible intervention combinations for a safe exit

#### Baseline scenario

Under the *baseline* scenario, 71, 77, and 89 out of the new 100 sample sets from the reduced parameter space (see methods and the new ranges in S2 Text) result in safe exits from the Zero-COVID policy for China, Shanghai, and Shiyan, respectively. We did not reduce the parameter space for Shenzhen, since its median mortality rate was already low (Fig. 3A and S2 Text).

The probability density curves of the median mortality rate in the reduced space are shown in Fig. S6 and the validations of the GP models are shown in Fig. S7. As expected, the resulted median mortality rates were all very close to the desired range (Fig. S6) and the fitted GP models can make accurate predictions (Fig. S7). The permutation importance of each intervention parameter in the reduced parameter space is shown in Fig. S8. The three most important parameters for China and Shiyan are still *ICU*, *Antiviral* and *ΔVac. 70above*, while those for Shanghai are *ICU*, *ΔVac. 20–59* and *ΔVac. 60–69*. We noticed that *ΔVac. 70above* is not among the top three for Shanghai, since the predefined ranges of the increase of the vaccine coverage for those above 70 years old are already very narrow (S2 Text). An interface to these full models is provided at https://canalcheng.shinyapps.io/COVID_exit/ and can be used to predict the median mortality rate of any combinations of intervention parameters interactively.

**Fig. 4:**
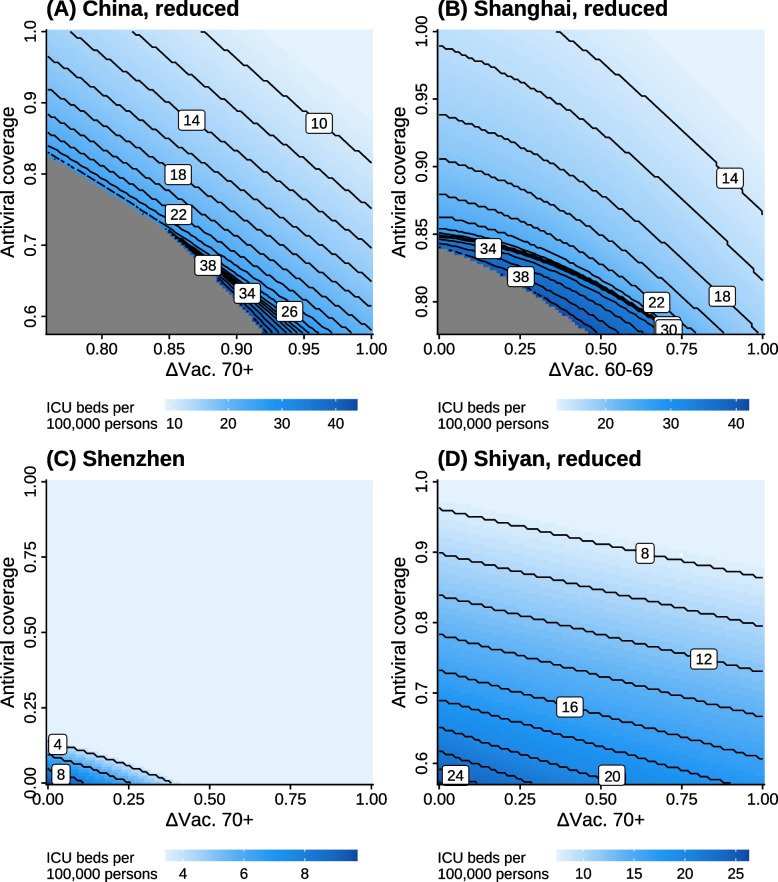
Feasible intervention combinations of the three most important intervention parameters by location under the *baseline* scenario. The color of a pixel shows the lowest number of ICU beds per 100,000 persons required for a safe exit, while the x- and y-axis show the other two most important intervention parameters. Black solid lines are the contour of the lowest number of ICU beds per 100,000 persons required for a safe exit. Note that the x- and y-axis and color schemes vary between panels

The simplified GP models fitted with only the three most important parameters still present reasonable predictive power on the out-of-bag samples (Fig. S9), with Pearson’s correlation coefficients of at least 0.93 across scenarios and locations. We used them to make predictions on a fine grid of the three most important parameters, and estimated the minimal number of ICU beds per 100,000 persons (colors of the pixels in Fig. 4) required for each combination of the other two important intervention parameters (x- and y-axis of Fig. 4) for visualizing the feasible region. There are clear tradeoffs between the three intervention parameters. As the value of one parameter increase, the minimal values of the other two required for a safe exit decrease. Reaching safe exits are possible, although extremely challenging for China, Shanghai, and Shiyan, which requires very high vaccine coverage, antiviral coverage, or number of ICU beds, or all three. However, it is relatively easy for Shenzhen, which only requires a 15% antiviral coverage, or a 41% increase in the vaccine coverage among the above 70 years old, or increasing ICU beds to 9.73 per 100,000 persons. Note that for Shanghai, a lower bound of 0.98 was set for *ΔVac. 70above* in the reduced space. Therefore, besides the three parameters presented in Fig. 4B, reaching a safe exit also requires *ΔVac. 70above* to be greater than 0.98 (S2 Text). Figure 4 can be used as a tool for identifying possible combinations of intervention parameters that result in safe exits for policy makers. Further considerations concerning the economic cost and societal impacts can be incorporated when making decisions.

#### Sensitivity analyses

The results under other scenarios are shown in S3 Text. When assuming a high VE (*optimistic VE* scenario), it is possible for Shenzhen to achieve a safe exit even with the current vaccine coverage, number of ICU beds per capita, and without antiviral treatment.

## Discussion

As COVID-19 is likely to become an endemic disease with an acceptable IFR, moving on from the Zero-COVID strategy was inevitable and is currently in progress. Hence, careful plans are needed to avoid hospital capacity strains and excessively high mortality rates. Although increasing the vaccine coverage of the elderly population, expanding healthcare and testing capacities, and stockpiling antiviral treatments have been proposed as possible pathways to accomplish this, it has not been studied quantitatively in order to determine thresholds of these interventions required to ensure a safe transition. We developed an individual-based model to examine the importance of various interventions and delineate the feasible intervention parameter space for a safe exit, defined as achieving a mortality rate lower than that of influenza, or 14.3 per 100,000 persons per year. The results show that the possibility of safe exits depend highly on the vaccine coverage of those above 70 years old, coverage of antiviral treatment, and the number of ICU beds per capita, as well as the vaccine effectiveness (VE), age structure, and vaccine coverage of the population.

Our results that achieving safe exits without stringent interventions are exceptionally challenging are consistent with previous modelling studies for the Omicron variant in China. In Cai et al. [5] which assumes a low reproductive number of 3.9, a high vaccine effectiveness as the *optimistic VE* scenario in our study, and no excessive mortality rate when hospitals are strained, maintaining the COVID-19 deaths below that of influenza would require that 100% of symptomatic cases receive antiviral treatment, regardless of their ascertainment status. In Leung et al.[7], which assumes a reproductive number of 8.3, a vaccine with higher effectiveness and a slower wane of the protection against hospitalizations and deaths than our *optimistic VE* scenario, having a 85% coverage for the fourth vaccine dose and a 60% antiviral treatment coverage still ends in a mortality rate more than 3 times the influenza mortality rate.

In the sensitivity analyses, we found that the impact of the proportion of symptomatic cases being rapid antigen tested (*75% testing* scenario) were insignificant under both the best- and worst-case intervention scenarios although it was more noticeable under the former than the latter. This inconsistency is likely due to the difference in antiviral coverage under these scenarios. Under the best-case intervention scenario, when all ascertained cases are treated by the antiviral drugs, a reduction in the number of cases due to decreased testing coverage leads to a decrease in the number of people treated and therefore an increased mortality rate; while under the worst-case intervention scenario, when antiviral treatment are not available, the impact of testing coverage is minimal. The impact of R_0_ on the mortality rate is insignificant (*R*_*0*_ = *5* or *R*_*0*_ = *10* scenarios). R_0_ affects the median mortality through affecting the transmission intensity. However, since the R_0_ values we examined here are already high, that is to say the proportions of individuals ultimately infected are already high, the relative increase in the incidence rate and therefore the mortality rate is low. The proportion of people being infected at the end of the simulation is 0.92 to 0.98 when R_0_ is set to 5, which only increased to 0.993 to 0.998 when R_0_ is set to 10. That relative susceptibility of children and adolescents and the relative infectivity of asymptomatic cases (*Lower Child. Sus.* and *Lower Asymp. Inf.* scenarios) have little impact on the mortality rate is not surprising, since they were used to estimate ${\beta }_{0}$, the transmission probability per contact. Their impacts on the transmission dynamics are cancelled out when the value of R_0_ is predefined. The impact of changes in the home contact rate after ascertainment on the mortality rate is limited (*No Self-Isolation* scenario), because only a very small proportion of cases were identified and self-isolated. The amount of increase in mortality rate when the required hospital beds are not available does not affect the mortality rate except for Shanghai under the worst-case scenario (5* and 2*Hosp. Mort. scenarios), since hospital beds are sufficient.

Our result that the number of ICU beds per capita, but not the number of hospital beds per capita, played an important role in determining the mortality rate is consistent with the conclusion of a previous study [5]. That study found that peak hospital bed demands are always below the number of hospital beds available for respiratory illness in China under various intervention scenarios, but the peak ICU bed demands are only lower than the current ICU capacity when the effective reproduction rate is low, the effectiveness and the coverage of the antiviral treatment are high, or heterologous booster with subunit vaccines were used. Our result that the coverage of antiviral treatment is among the most important interventions in determining the morality rate is also consistent with theirs, although our assumption about who is eligible for antiviral treatment differs from theirs. They assumed that all symptomatic cases over 12 years old are eligible for antiviral treatments, regardless of their ascertainment status, while we made a more realistic assumption that only detected case will be treated.

Our study has several limitations. First, our definition of a safe exit as having a mortality rate lower than that of influenza is arbitrary, although influenza is the disease that COVID-19 is most often compared with. However, the method developed here could be easily adapted to examine the results for other definitions of safe exits. Secondly, due the lack of individual-based census data required by the population synthesis algorithms to generate household structure [34, 35], we were not able to include household structure in the synthetic population. We assumed that the cases isolated at home still had contact with anyone in the synthetic population, but at rates estimated in the home setting, not the sum across all settings (Fig. S4). This assumption may result in an overestimation of the case count since cases have access to more susceptibles under this assumption which makes it harder to run out of susceptibles and thereby end the transmission. However, the impact of not simulating the household structure on the mortality rate should be negligible. As in the *75% testing* and *no self-isolation* scenarios, since the ascertained cases consists only a very small proportion of all cases, their impact on the overall transmission is limited. Furthermore, we did not consider the impact of different demography on contact patterns, since according to our preliminary examination, its impact on the distribution of mortality rate is negligible. Thirdly, our assumption that the social mixing pattern would return to its pre-pandemic levels immediately after the end of the Zero-COVID policy may lead to overestimation of the mortality rates, since according to Google Mobility Trends, the amount of time people spend in residential areas is still higher, while the number of visitors to workplaces, transit stations, and retails are still lower than the pre-pandemic level long after the interventions are lifted [36]. However, since we calibrated to the model to have a predefined R_0_, a lower contact rate should not affect the final incidence and mortality rates, since the estimated ${\beta }_{0}$ will increase in this case to match the predefined R_0_. Fourthly, we only examined a limited set of interventions because of their feasibility in China. For example, we left out possible effective interventions such as using subunit vaccines as the third or fourth dose after two doses of inactivated vaccines as the primary vaccinations, because they are still not available in China. Lastly, our study only focuses on China and the results might be different for other places and countries. However, the analytical framework can be modified easily to examine the results for other places and other diseases as well.

## Conclusions

In conclusion, through the individual-base model and the analytical framework we developed, we identified the importance of increasing vaccine coverage in the older age groups, antiviral coverage and the number of ICU beds per capita in determining the median mortality rate and provided combinations of them that can result in safe exits for China as a whole and three representative cities. The exact thresholds for these interventions ensuring safe exits depends on the vaccine effectiveness, and the age structure and current vaccine coverage of the studied population. The model developed here can be easily extended to other definitions of safe exits and applied to other locations for developing transition plans based on local context.

## Supplementary Information

**Additional file 1.**

## Data Availability

The code and data to reproduce the results of the article are available at https://github.com/qu-cheng/COVID_exit.

## Abbreviations

GP: Gaussian process
IBM: Individual-based model
IFR: Infection fatality rate
VE: Vaccine effectiveness

## References

[1] Chen JM, Chen YQ. China can prepare to end its zero-COVID policy. Nature Medicine. 2022;28(6):1104–5. 10.1038/s41591-022-01794-3 35383312

[2] Skegg DC, Hill PC (2021). Defining covid-19 elimination. In., vol. 374: British Medical Journal Publishing Group.

[3] Lewnard JA, Hong VX, Patel MM, Kahn R, Lipsitch M, Tartof SY. Clinical outcomes associated with SARS-CoV-2 Omicron (B. 1.1. 529) variant and BA. 1/BA. 1.1 or BA. 2 subvariant infection in southern California. Nat Med. 2022;28(9):1933–43. 10.1038/s41591-022-01887-z PMC10208005 35675841

[4] Why insist on dynamic Zero-COVID? [http://www.gov.cn/fuwu/2022-04/29/content_5688064.htm]

[5] Cai J, Deng X, Yang J, Sun K, Liu H, Chen Z, Peng C, Chen X, Wu Q, Zou J, Sun R. Modeling transmission of SARS-CoV-2 omicron in China. Nat Med. 2022;28(7):1468–75. 10.1038/s41591-022-01855-7 PMC9307473 35537471

[6] Wang Y, Sun K, Feng Z, Yi L, Wu Y, Liu H, et al. Assessing the feasibility of sustaining SARS-CoV-2 local containment in China in the era of highly transmissible variants. BMC Med. 2022;20(1):442. 10.1186/s12916-022-02640-6 PMC9666984 36380354

[7] Leung K, Leung GM, Wu J. Modelling the adjustment of COVID-19 response and exit from dynamic zero-COVID in China [Internet]. medRxiv [Preprint]. 2022. p. 42. [cited 2022 Dec 28]. Available from: https://www.medrxiv.org/content/10.1101/2022.12.14.22283460v1.

[8] Li J, Chen Y, Wang X, Yu H (2021). Influenza-associated disease burden in mainland China: a systematic review and meta-analysis. Sci Rep.

[9] Mossong J, Hens N, Jit M, Beutels P, Auranen K, Mikolajczyk R, Massari M, Salmaso S, Tomba GS, Wallinga J (2008). Social contacts and mixing patterns relevant to the spread of infectious diseases. PLoS Med.

[10] Zhang J, Klepac P, Read JM, Rosello A, Wang X, Lai S, Li M, Song Y, Wei Q, Jiang H (2019). Patterns of human social contact and contact with animals in Shanghai, China. Sci Rep.

[11] Wang Y, Tian H, Zhang L, Zhang M, Guo D, Wu W, Zhang X, Kan GL, Jia L, Huo D (2020). Reduction of secondary transmission of SARS-CoV-2 in households by face mask use, disinfection and social distancing: a cohort study in Beijing, China. BMJ Glob Health.

[12] Chen X, Chen Z, Azman AS, Deng X, Sun R, Zhao Z, Zheng N, Chen X, Lu W, Zhuang T (2021). Serological evidence of human infection with SARS-CoV-2: a systematic review and meta-analysis. Lancet Glob Health.

[13] COVID-19 Dashboard by the Center for Systems Science and Engineering (CSSE) at Johns Hopkins University (JHU). https://coronavirus.jhu.edu/map.html.

[14] Epidemiological update on COVID-19. http://wjw.wuhan.gov.cn/ztzl_28/fk/yqtb/.

[15] Wölfl-Duchek M, Bergmann F, Jorda A, Weber M, Müller M, Seitz T, Zoufaly A, Strassl R, Zeitlinger M, Herkner H (2022). Sensitivity and specificity of SARS-CoV-2 rapid antigen detection tests using oral, anterior nasal, and nasopharyngeal swabs: a diagnostic accuracy study. Microbiol Spectr.

[16] Ma Y, Xu S, An Q, Qin M, Li S, Lu K, Li J, Lei L, He L, Yu H (2022). Coronavirus disease 2019 epidemic prediction in Shanghai under the “dynamic zero-COVID policy” using time-dependent SEAIQR model. J Biosaf Biosecur.

[17] Chen X, Yan X, Sun K, Zheng N, Sun R, Zhou J, et al. Estimation of disease burden and clinical severity of COVID-19 caused by Omicron BA. 2 in Shanghai, February-June 2022. Emerg Microbes Infect. 2022;11(1):2800–7. 10.1080/22221751.2022.2128435 PMC9683067 36205530

[18] Kerr CC, Stuart RM, Mistry D, Abeysuriya RG, Rosenfeld K, Hart GR, Núñez RC, Cohen JA, Selvaraj P, Hagedorn B (2021). Covasim: An agent-based model of COVID-19 dynamics and interventions. PLoS Comput Biol.

[19] Tan M, Wang Y, Luo L, Hu J (2021). How the public used face masks in China during the coronavirus disease pandemic: a survey study. Int J Nurs Stud.

[20] Leech G, Rogers-Smith C, Monrad JT, Sandbrink JB, Snodin B, Zinkov R, Rader B, Brownstein JS, Gal Y, Bhatt S (2022). Mask wearing in community settings reduces SARS-CoV-2 transmission. Proc Natl Acad Sci.

[21] Carbonell R, Urgelés S, Rodríguez A, Bodí M, Martín-Loeches I, Solé-Violán J, Díaz E, Gómez J, Trefler S, Vallverdú M (2021). Mortality comparison between the first and second/third waves among 3,795 critical COVID-19 patients with pneumonia admitted to the ICU: A multicentre retrospective cohort study. Lancet Reg Health Eur..

[22] Auld SC, Caridi-Scheible M, Blum JM, Robichaux C, Kraft C, Jacob JT, Jabaley CS, Carpenter D, Kaplow R, Hernandez-Romieu AC (2020). ICU and Ventilator Mortality Among Critically Ill Adults With Coronavirus Disease 2019*. Crit Care Med.

[23] Armstrong R, Kane A, Kursumovic E, Oglesby F, Cook TM (2021). Mortality in patients admitted to intensive care with COVID-19: an updated systematic review and meta-analysis of observational studies. Anaesthesia.

[24] Intensive care beds per 100,000, 2020. https://ourworldindata.org/grapher/intensive-care-beds-per-100000?tab=table&time=latest.

[25] Hospital beds (per 1,000 people). https://data.worldbank.org/indicator/SH.MED.BEDS.ZS?most_recent_value_desc=true.

[26] McMenamin ME, Nealon J, Lin Y, Wong JY, Cheung JK, Lau EH, et al. Vaccine effectiveness of one, two, and three doses of BNT162b2 and CoronaVac against COVID-19 in Hong Kong: a population-based observational study. Lancet Infect Dis. 2022;22(10):1435–43. 10.1016/S1473-3099(22)00345-0 PMC9286709 35850128

[27] Shao W, Chen X, Zheng C, Liu H, Wang G, Zhang B, Li Z, Zhang W (2022). Effectiveness of COVID-19 vaccines against SARS-CoV-2 variants of concern in real-world: a literature review and meta-analysis. Emerg Microbes Infect.

[28] Florian A (1992). An efficient sampling scheme: updated latin hypercube sampling. Probab Eng Mech.

[29] Wang J (2020). An intuitive tutorial to Gaussian processes regression. arXiv preprint arXiv:200910862.

[30] Domingo D (2019). Gaussian process emulation: theory and applications to the problem of past climate reconstruction. University of Leeds.

[31] Greenwell BM, Boehmke BC, Gray B (2020). Variable Importance Plots-An Introduction to the vip Package. R J.

[32] Office of the Leading Group of the State Council for the Seventh National Population Census: Tabulation on 2020 China Population Census by County. 1st ed. Beijing: China Statistics Press; 2022.

[33] Levin AT, Hanage WP, Owusu-Boaitey N, Cochran KB, Walsh SP, Meyerowitz-Katz G (2020). Assessing the age specificity of infection fatality rates for COVID-19: systematic review, meta-analysis, and public policy implications. Eur J Epidemiol.

[34] Ye X, Konduri K, Pendyala RM, Sana B, Waddell P (2009). A methodology to match distributions of both household and person attributes in the generation of synthetic populations. 88th Annual Meeting of the transportation research Board.

[35] Huang Z, Williamson P (2001). A comparison of synthetic reconstruction and combinatorial optimisation approaches to the creation of small-area microdata.

[36] COVID-19: Google Mobility Trends. https://ourworldindata.org/covid-google-mobility-trends.

