# Supplementary material for: Feasible intervention combinations for achieving a safe exit of the Zero-COVID policy in China and its determinants: an individual-based model study
Source: BMC Infect Dis. 2023 Jun 12;23:390. doi: 10.1186/s12879-023-08382-x (PMC10258473; doi:10.1186/s12879-023-08382-x)
Supplement: Supplementary file 1 — Additional file 1. [file 12879_2023_8382_MOESM1_ESM.pdf]

Supplementary Information for

Feasible intervention combinations for achieving a safe exit of the dynamic Zero-COVID policy in China and its determinants: an individual-based model study

Qu Cheng*, Xingjie Hao, Degang Wu, Qi Wang, Robert C. Spear, Sheng Wei*

* Correspondence to: Qu Cheng: chengqu@hust.edu.cn; Sheng Wei: shengwei@hust.edu.cn

Table of contents

S1 Text. Method for estimating the force of infection
Table ST1.1. Scaling parameters moderating the force of infection
Table ST1.2. Decreasing vaccine effectiveness (VE) due to waning
S2 Text. Methods and results for the reduced parameter space
Table ST2.1. Intervention parameter values under the best- and worst-case scenarios
Table ST2.2. Lower bounds of the intervention parameter values for the reduced parameter space by location and sensitivity scenarios
Fig. ST2.1. Probability of safe exit when changing one parameter at a time from its worst-case to best-case scenario value while keeping the other parameters at their best-case scenario values
S3 Text. Results for the sensitivity analyses
Fig. ST3.1. Distributions of the mortality rates under different sensitivity analysis scenarios for the best-case and worst-case intervention parameter values by location
Fig. ST3.2 Results of the 100 samples from the full parameter space by location and sensitivity scenario
Fig. ST3.3 Median mortality rates by location and sensitivity scenario observed from simulations and predicted by Gaussian process models with samples from the full parameter space
Fig. ST3.4 Feasible intervention combinations of the three most important intervention parameters by location and intervention scenarios

Fig. S1 Proportion of population in each age group for each location used for generating the synthetic population
Fig. S2 Age-specific vaccine coverage at different locations used for generating the synthetic population
Fig. S3 Simulated mortality rate for three random sets of intervention parameters by the number of agents for China
Fig. S4 Smoothed number of contacts between age groups at (A) all settings and (B) only home settings
Fig. S5 Median mortality rates by location and sensitivity scenario observed from simulations and predicted by Gaussian process models with samples from the full parameter space under the baseline scenario
Fig. S6 Median mortality rates by location and sensitivity scenarios for the 100 random samples from the reduced intervention parameter space and their probability density curves
Fig. S7 Median mortality rates by locations and sensitivity scenarios observed from simulations and predicted by Gaussian process models with samples from the reduced parameter space
Fig. S8 Permutation importance of each intervention parameter for predicting the median mortality rate (colored bars) in the reduced parameter space
Fig. S9 Median mortality rates by locations and sensitivity scenarios observed from simulations and predicted by Gaussian process models fitted with only the three most important parameters

Table S1. Number of hospital and ICU beds per capita for different locations
Table S2. Parameter values for the sensitivity analysis
Table S3. Age-dependent transition rates when not vaccinated
Table S4. Reduction in the transition rates by vaccine dose
Table S5. The distribution of waiting times between states

S1 Text. Method for estimating the force of infection

The force of infection was estimated as $\lambda_{i,t} = 0 \cdot 75^{p_m} \Sigma_{j=1}^{I_t} \beta_0 C_{a_i,a_j} \rho_{V_i} \rho_{V_j} \rho_{a_i} \rho_{s_j} \rho_{p_j} \Delta t$, where $0 \cdot 75^{p_m}$ represents the reduced transmission for a given mask coverage p_m according to a community study(1) (See *Interventions* in the main text for details); I_t is the total number of infectious individuals in the synthetic population at time *t*; β_0 is the transmission probability per contact and was calibrated to have a R_0 of 7 for the Omicron variant according to previous studies (R_0 was set to 5 and 10 in the sensitivity analyses, *R_0 = 5* and *R_0 = 10* scenarios in Table S2);(2-5) C_{a_i,a_j} is the average number of contacts per day between the susceptible *i* and the case *j* (whose ages are a_i and a_j, respectively) at *all* locations when *j* is not identified, or only at *home* setting when *j* is identified and self-isolated at home; (6) the ρs are the scaling parameters which vary between zero and one and thereby reduce the force of infection depending on the vaccination status, age groups, symptoms, and ascertainment status of the cases or the susceptibles (Table ST1.1); and Δt = 1/6 day is the time step of the simulation. The vaccine induced moderator of infection risk and onward transmission were represented by $\rho_{V_i}$and ρ_{V_j}, respectively. Therefore, 1-ρ_{V_i} and 1-ρ_{V_j} represents the VEs against infection and onward transmission. The VEs in the baseline scenario were estimated as the mean of the optimistic and pessimistic scenarios from (5). We assumed that the vaccine-induced immunity against infection, symptom, hospitalization, ICU care and death wanes at a constant rate over time until reaching a certain percentage of the original VEs (Table ST1.2) according to a meta-analysis,(7) while that against onward transmission did not wane since it was already very low. We ran further sensitivity analyses to account for uncertainties in the current knowledge about the ρs (Table ST1.1and Table S2).

Table ST1.1. Scaling parameters moderating the force of infection

**Parameters**	**Definition**	**Value**	**Sensitivity analysis** (also see Table S2)
ρ_{V_i}	Vaccine-induced moderator of infection risk	0·969, 0·930, and 0·869 for the first, second, and third doses, respectively	***Optimistic VE:*** 0·994, 0·952, and 0·908 for the first, second, and third doses, respectively; ***Pessimistic VE:*** 0·944, 0·909, and 0·83 for the first, second, and third doses, respectively (5).
ρ_{V_j}	Vaccine-induced moderator of onward transmission	1, 1, and 0·947 for the first, second, and third doses, respectively	***Optimistic VE:*** 1 for all doses; ***Pessimistic VE:*** 1, 1, and 0·894 for the first, second, and third doses, respectively(5)
ρ_{a_i}	Relative susceptibility of children (<10 years old) and adolescents (10-19 years old) when compared with adults	**Children:** 1 **Adolescents:** 1(8, 9)	***Lower Child. Sus.*** **scenario:** **Children:** 0·52 **Adolescents:** 0·72(10)
ρ_{s_j}	Relative infectiousness of asymptomatic cases when compared with presymptomatic or symptomatic cases	1(9)	***Lower Asymp. Inf.*** **scenario:** 0·3(11-13)
ρ_{p_j}	Relative contact rate once identified and isolated	0·2(14)	***No Self-Isolation*** **scenario:** 1

Table ST1.2. Decreasing vaccine effectiveness (VE) due to waning (15)

**Dose**	**Protection against infection and symptomatic cases**		**Probability of becoming a severe/critical case from a mild case**		**Probability of death from a severe/critical case**	
	**Decreasing rate (%/month)**	**Minimum % of the original VE**	**Decreasing rate (%/month)**	**Minimum % of the original VE**	**Decreasing rate (%/month)**	**Minimum % of the original VE**
**1 & 2**	7.7	13	2.6	88	2.6	90
**3**	7.9	62	5.7	95	5.7	96.6

S2 Text. Methods and results for the reduced parameter space

To identify more promising ranges of each intervention parameter likely to result in morality rates closer to the desired mortality rate, we changed each value to be between its worst- and best-case scenario values (Table ST2.1) while keeping the other parameters at their best-case scenario values. The value found when the percentage of repetitions having a desired mortality rate first exceeding 95 was set as the lower bound of the reduced range for this parameter.

Only the ranges of ICU, ΔVac. 70above, or Antiviral were cut (Table ST2.2), because they are the only parameters that were able to affect the probability of safe exits (Fig. ST2.1). We did not cut the range for Shenzhen under all scenarios, and Shiyan under the *optimistic VE* scenario, since they already had high chances of resulting in safe exits in the full space.

Table ST2.1. Intervention parameter values under the best- and worst-case scenarios

**Intervention parameter**	**Best-case**	**Worst-case**			
		**China**	**Shanghai**	**Shenzhen**	**Shiyan**
Mask	1	0	0	0	0
Hospital (per 1,000 persons)	14.4	5.06	5.78	3.27	6.82
ICU (per 100,000 persons)	48	4.37	6.14	3.42	5.13
ΔVac. 0-19	1	0	0	0	0
ΔVac. 20-59	1	0	0	0	0
ΔVac. 60-69	1	0	0	0	0
ΔVac. 70above	1	0	0	0	0
Antiviral	1	0	0	0	0

Table ST2.2. Lower bounds of the intervention parameter values for the reduced parameter space by location and sensitivity scenarios. Empty cell means that the range is not reduced.

**Intervention parameter**	ICU (per 100,000 persons)	ΔVac. 70above	Antiviral
China, *baseline*	8.33	0.760	0.577
Shanghai, *baseline*	12.5	0.979	0.777
Shiyan, *baseline*	7.38		0.572
China, *optimistic VE*		0.330	
Shanghai, *optimistic VE*		0.793	

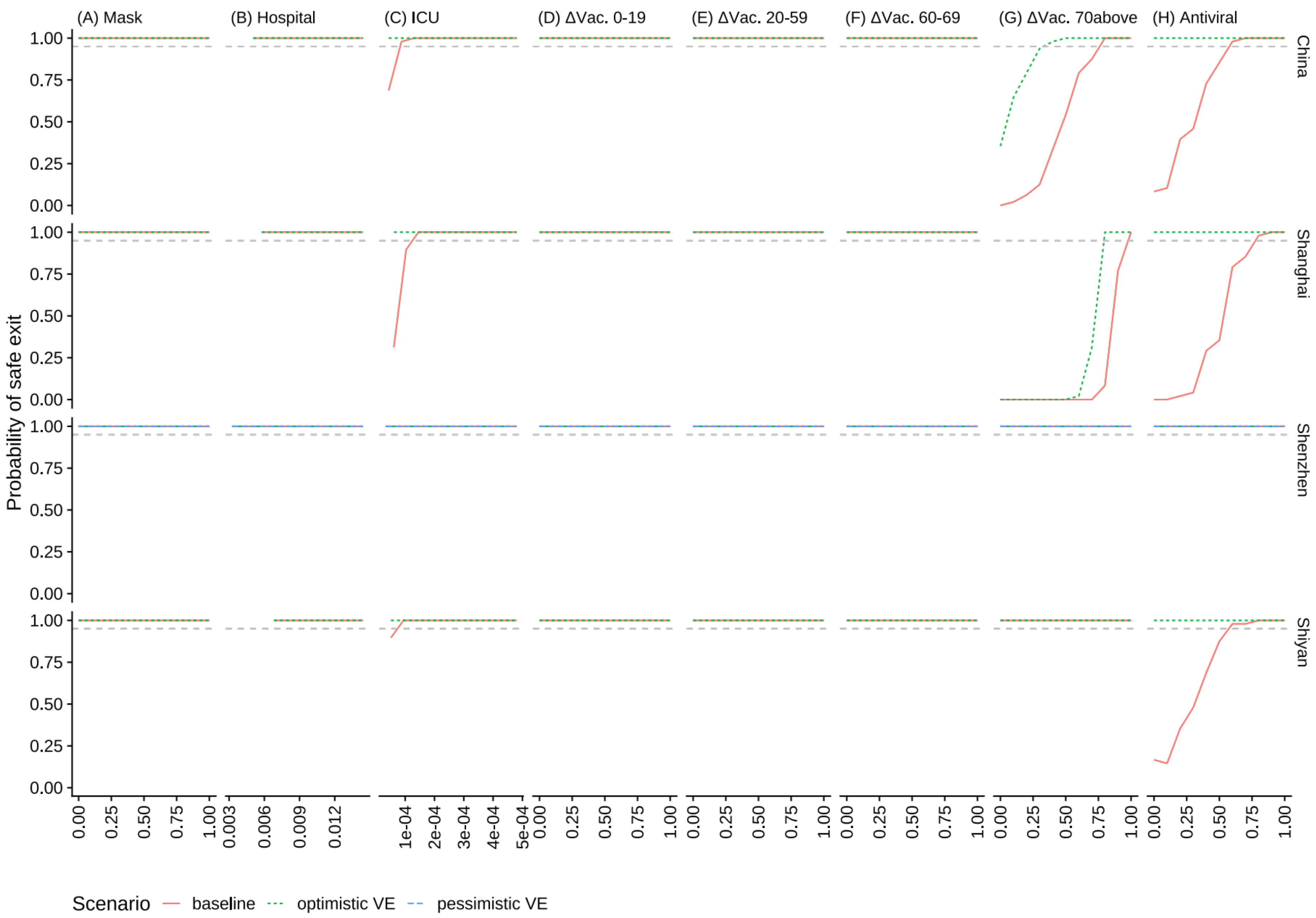

Fig. ST2.1 Probability of safe exit when changing one parameter at a time from its worst-case to best-case scenario value while keeping the other parameters at their best-case scenario values. Columns show the parameter being changed and rows show the scenarios examined. Horizontal dashed lines represent the safe exit probability of 0.95.

S3 Text. Results for the sensitivity analyses

Possibility of a safe exit. We examined the distribution of the mortality rate under different sensitivity scenarios (Fig. ST3.1). When compared the *75% testing* with the *baseline* scenario, the mortality rate increased marginally, but insignificantly, under the best-case intervention scenario, while almost no impacts were observed under the worst-case intervention scenario. Assuming a lower basic reproductive number ($R_0 = 5$ scenario), or higher ($R_0 = 10$ scenario), has almost no influence in the mortality rate under both scenarios. The impacts of the vaccine effectiveness, however, are remarkable with a higher VE (*optimistic VE* scenario) resulting in significantly lower and a lower VE (*pessimistic VE* scenario) significantly higher median mortality rates when compared with the *baseline* scenario. The relative susceptibility of children and adolescents to adults (*Lower Child. Sus.* scenario), relative infectivity of asymptomatic cases to presymptomatic and symptomatic cases (*Lower Asymp. Inf.* scenario), reduction in home contact rate after detection (*No Self-Isolation* scenario), and changes in mortality rate when having no access to hospital beds (*5*Hosp. Mort.* and *2*Hosp. Mort.* scenarios) had no significant impacts on the median mortality rate. Based on these results, the *optimistic VE* scenario for China, Shanghai and Shiyan, and both the *optimistic VE* and *pessimistic VE* scenarios for Shenzhen were included in further analyses, since they resulted in median mortality rates in the desired range and differ significantly from the *baseline* scenario.

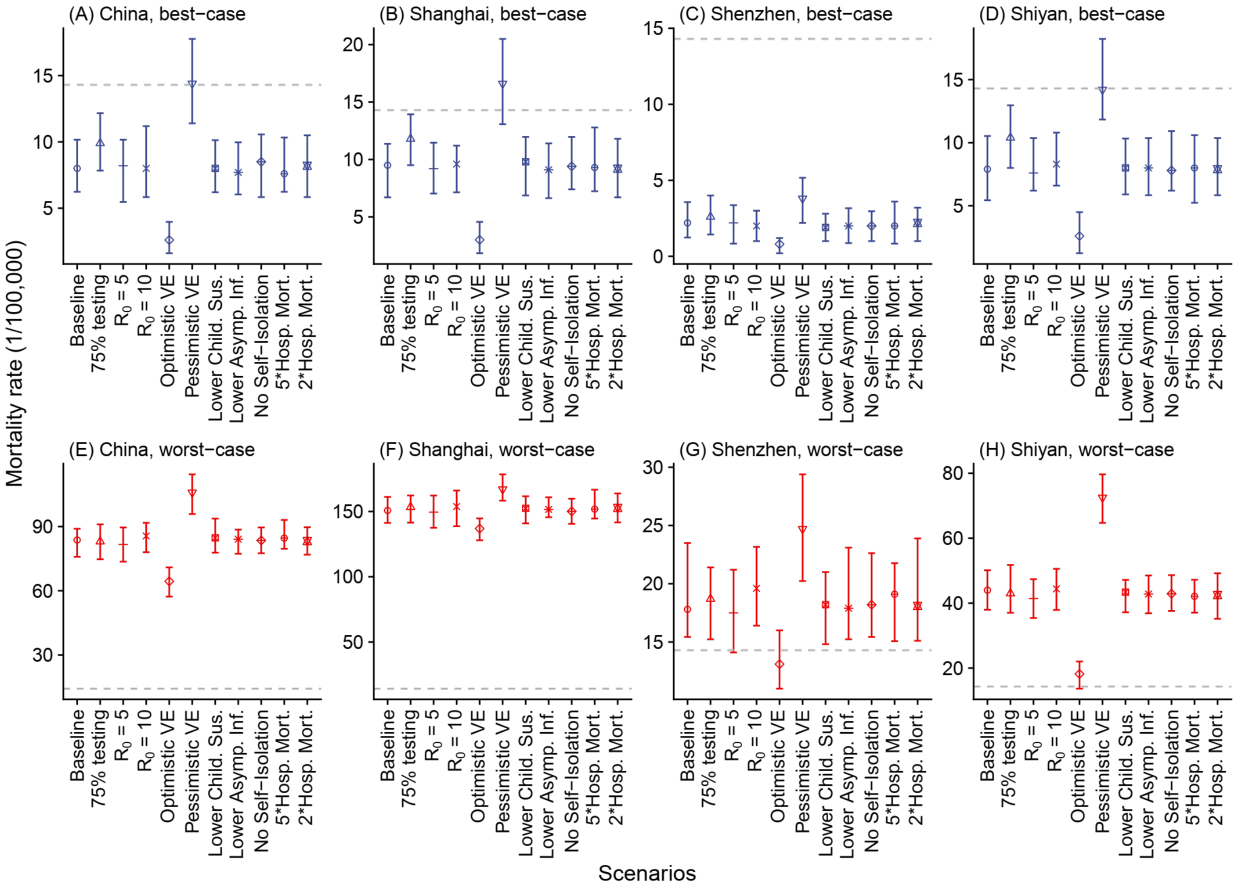

Fig. ST3.1. Distributions of the mortality rates under different sensitivity analysis scenarios (x-axis) for the best-case (first row) and worst-case (second row) intervention parameter values by location (columns). Symbols in each panel represent the median, while the error bars represent the 2·5 to 97·5 percentile of the mortality rate of the 48 repetitions. The gray dashed lines represent the annual mortality rate of influenza as 14·3 per 100,000 persons.

Importance of each intervention in determining the mortality rate. Under the *optimistic VE* scenario, 37, 11, 100 and 95 out of the 100 random samples from the full parameter space result in safe exits for China, Shanghai, Shenzhen, and Shiyan, respectively; with average median mortality rates of 18.3, 44.9, 3.67, and 8.17 per 100,000 persons (Fig. ST3.2A). Under the *pessimistic VE* scenario, we only examined the results for Shenzhen, since it was only possible for Shenzhen to safely exit the Zero-COVID policy under this scenario. For other locations, safe exits are impossible even under the base-case intervention scenario. For Shenzhen, 95 out of 100 random samples achieve median mortality rates lower than that of influenza, and the average median mortality rate was 6.12 per 100,000 persons. Results from the 10-fold cross-validation suggest that the fitted Gaussian process emulators have strong and robust predictive power, even on the out-of-bag samples that were not used in training the model (Fig. ST3.3). Therefore, we used them to examine the importance of each intervention in determining the mortality rate. The results show that, the same as under the *baseline*

scenario, for all locations, Hospital and ΔVac. 0-19 are the least important intervention parameters in determining the mortality rate, while ΔVac. 70above, Antiviral, and ICU are the most important parameters (Fig. ST3.2B).

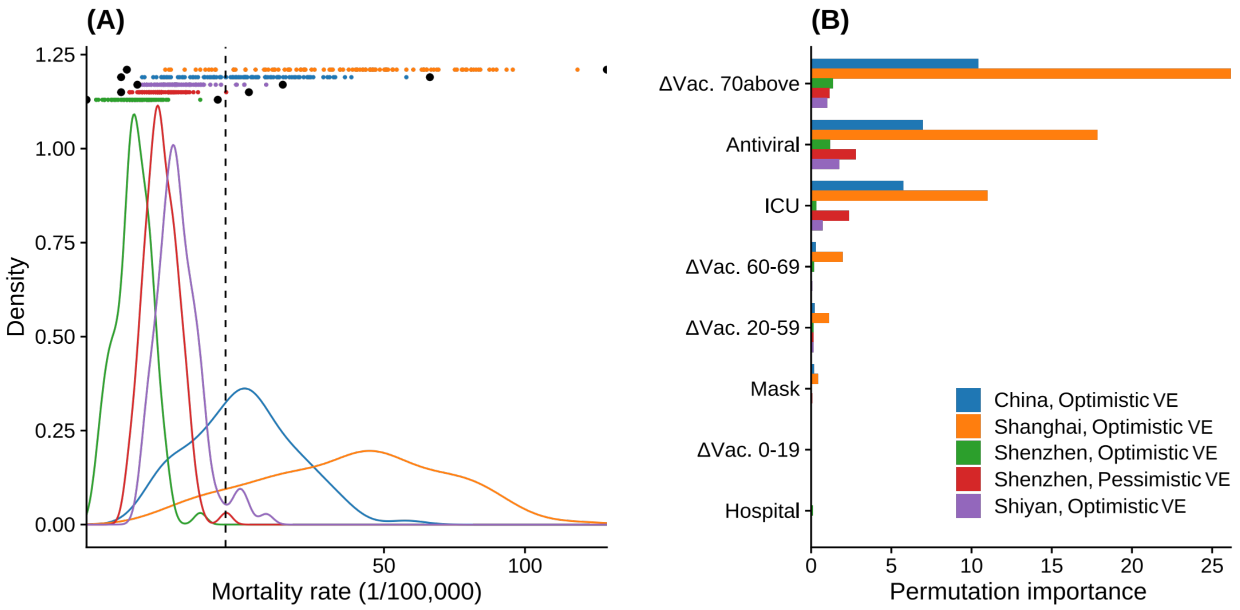

Fig. ST3.2 Results of the 100 samples from the full parameter space by location and sensitivity scenario. (A) Median mortality rates (colored dots) and their probability density distributions (colored curves) from the simulations. Black dots on the top of the panel show the median mortality rate from the simulations using the best-case (the dot on the left) and the worst-case scenario (the dot on the right) parameter values (Table ST1.1). Vertical dashed line shows the mortality rate of influenza (14.3 per 100,000 persons). (B) Permutation importance of each intervention parameter for predicting the median mortality rate (colored bars). The permutation importance of a parameter represents the amount of increase in the root mean squared prediction error when the randomly shuffled values of this parameter, together with the unchanged values of the other parameters, were used to make the prediction.

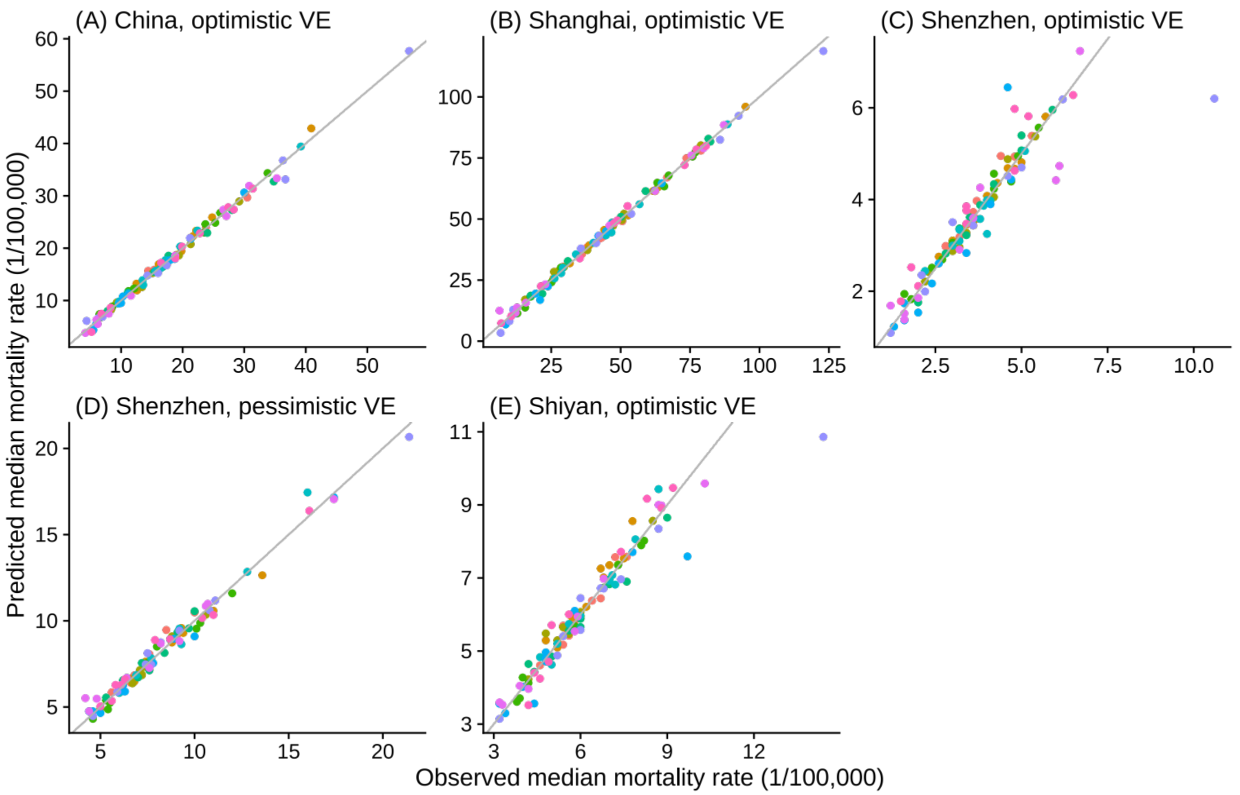

Fig. ST3.3 Median mortality rates by location and sensitivity scenario observed from simulations and predicted by Gaussian process models with samples from the full parameter space with a 10-fold cross-validation design. Different colors represent samples from different folds. The gray line in each panel represents the line of Y = X.

Feasible intervention combinations for a safe exit. In the reduced parameter space (see methods and the new ranges in S2 Text), under the *optimistic VE* scenario, 57 and 46 out of the new 100 sample sets result in safe exits from the Zero-COVID policy for China and Shanghai, respectively. For both locations, *ICU*, *Antiviral* and *ΔVac. 70above* are the most important three parameters (Fig. S8) and were used to fit the simplified GP models. The validation of the simplified models were shown in Fig. S9, with Pearson's correlation coefficients of at least 0.93 across scenarios and locations. We used them to make predictions on a fine grid of the three most important parameters, and estimated the minimal number of ICU beds per 100,000 persons (colors of the pixels in Fig. ST3.4) required for each combination of the other two important intervention parameters (x- and y-axis of Fig. ST3.4) for visualizing the feasible region. There are clear tradeoffs between the three intervention parameters. As the value of one parameter increase, the minimal values of the other two required for a safe exit decrease.

Reaching safe exits are possible, although extremely challenging for China and Shanghai, even under the *optimistic VE* scenario, which requires very high vaccine coverage, antiviral coverage, or number of ICU beds, or all three. However, it is always possible for Shenzhen under the *optimistic VE* scenario, but still requires high public health resource investments under the *pessimistic VE* scenario. For Shiyan under the *optimistic VE* scenario, reaching a safe exit requires a 62.8% antiviral coverage, or at least 57.2% antiviral coverage, together with a 26% increase in the vaccine coverage among the above 70 years old, or increasing ICU beds to 6.86 per 100,000 persons.

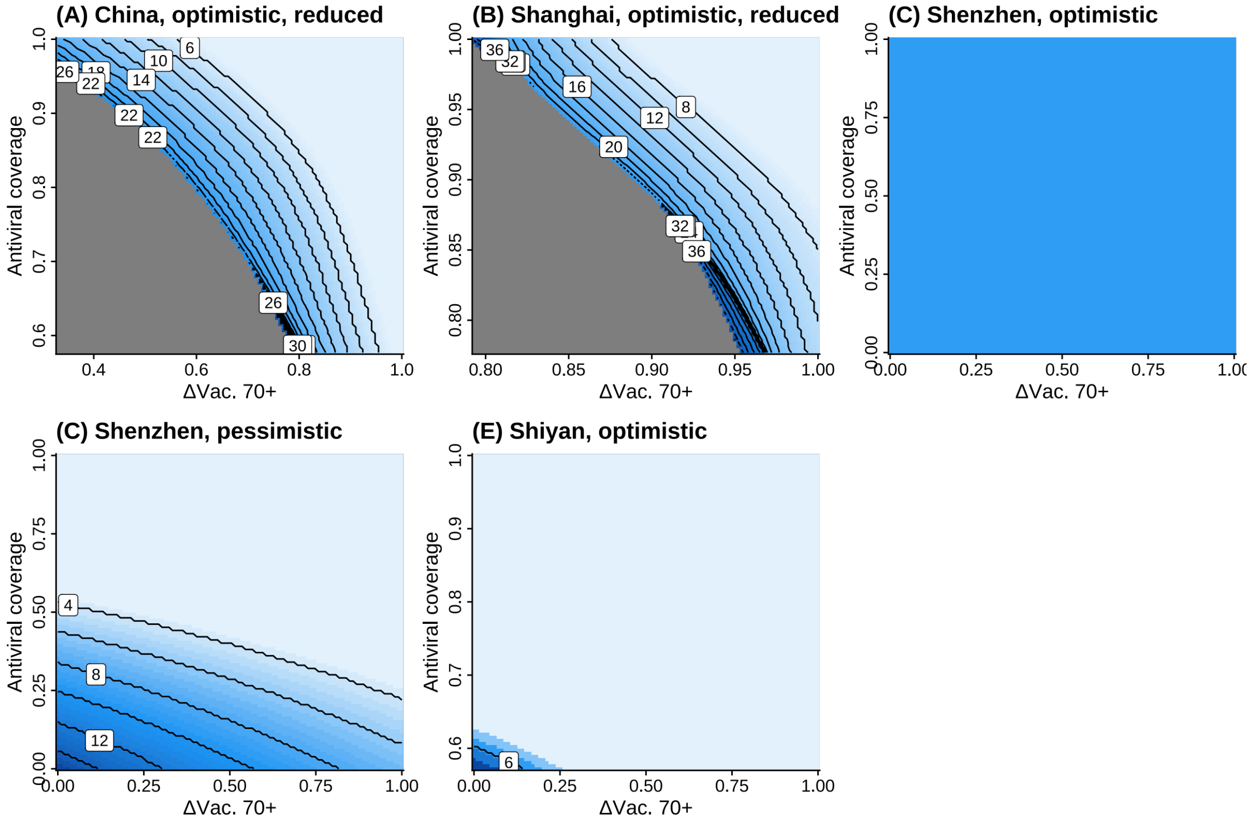

Fig. ST3.4 Feasible intervention combinations of the three most important intervention parameters by location and intervention scenarios. The color of a pixel shows the lowest number of ICU beds per 100,000 persons required for a safe exit, while the x- and y-axis show the other two most important intervention parameters. Note that the x- and y-axis and color schemes vary between panels.

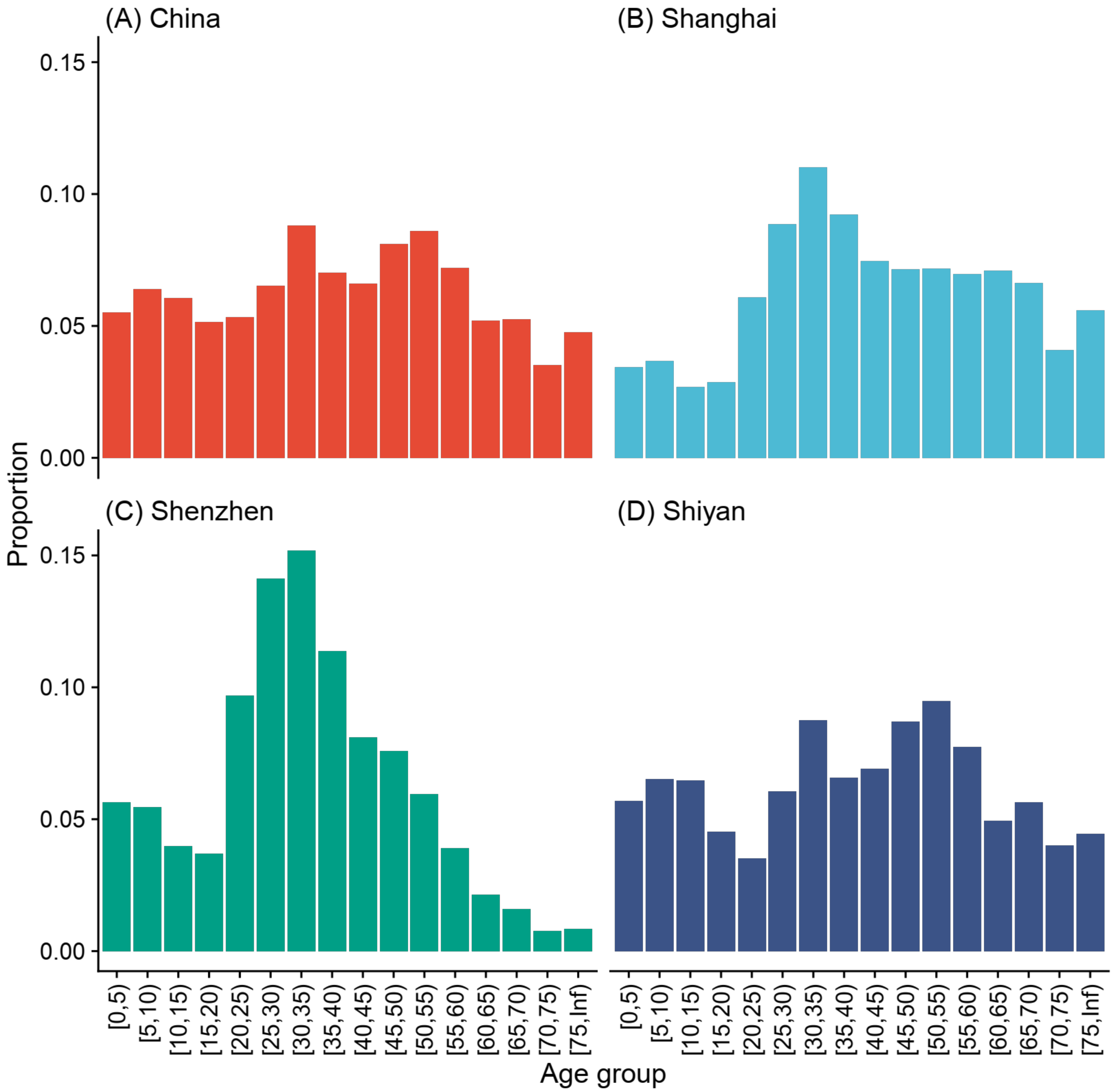

Fig. S1 Proportion of population in each age group for each location used for generating the synthetic population. The data were obtained from (16).

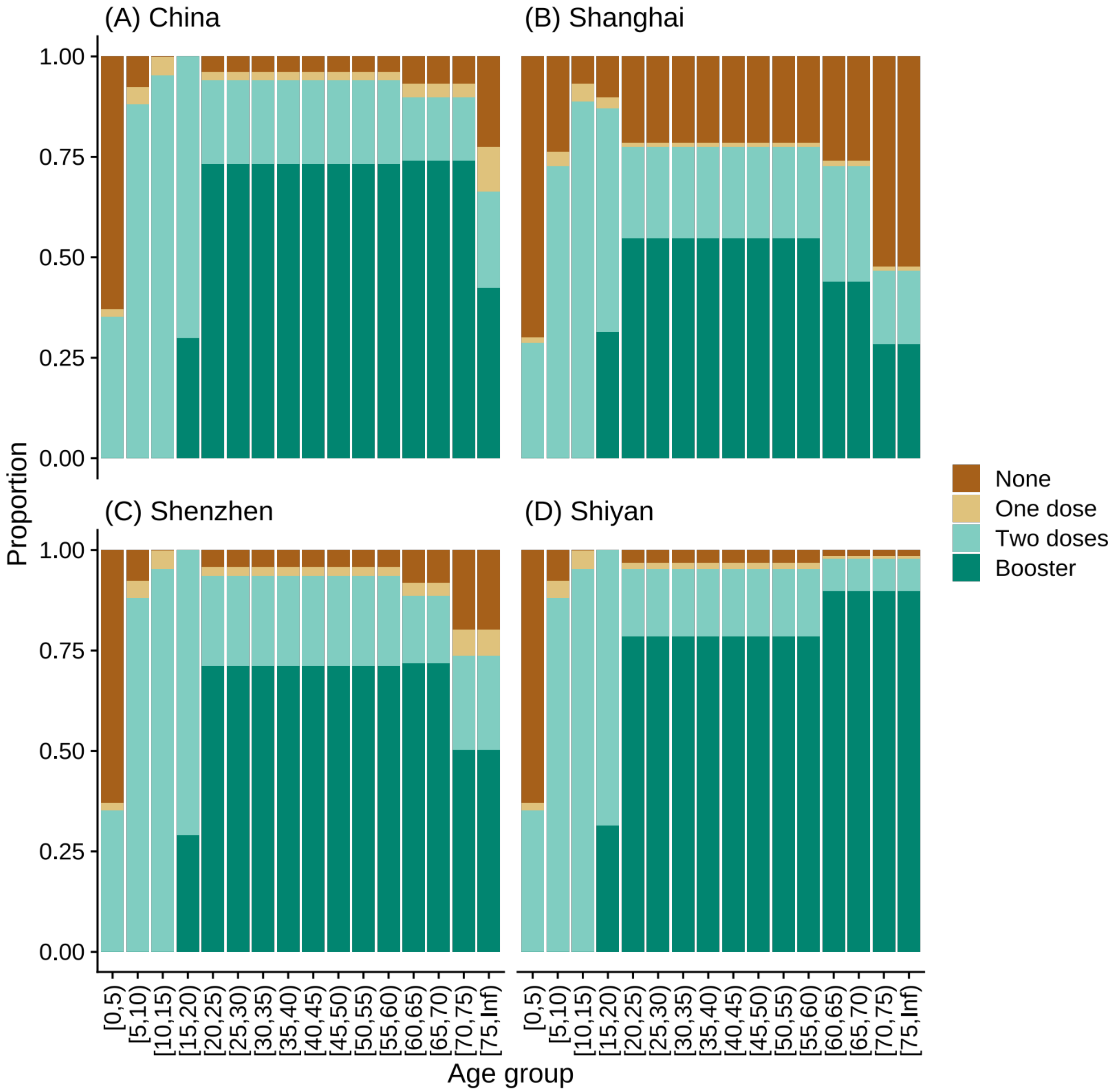

Fig. S2 Age-specific vaccine coverage at different locations used for generating the synthetic population (5, 17-20).

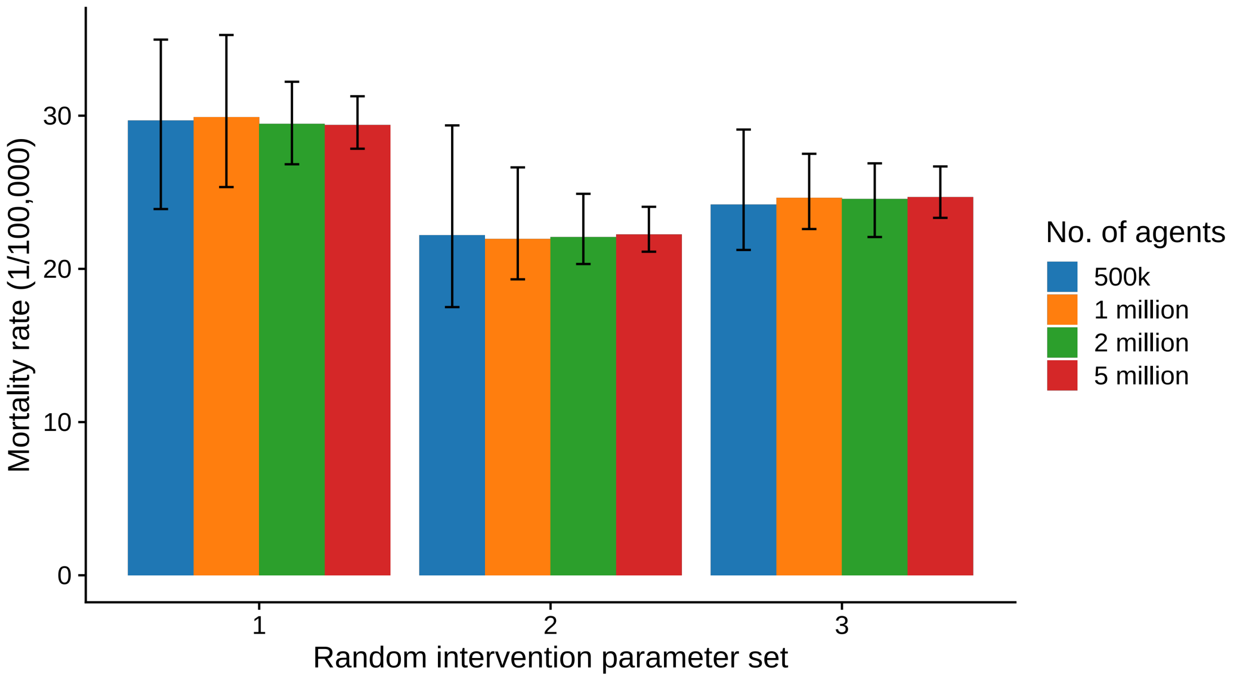

Fig. S3 Simulated mortality rate for three random sets of intervention parameters (x-axis) by the number of agents (colors) for China. Bars represent the median while error bars represent the 95% confidence intervals of mortality rates of the 48 repetitions.

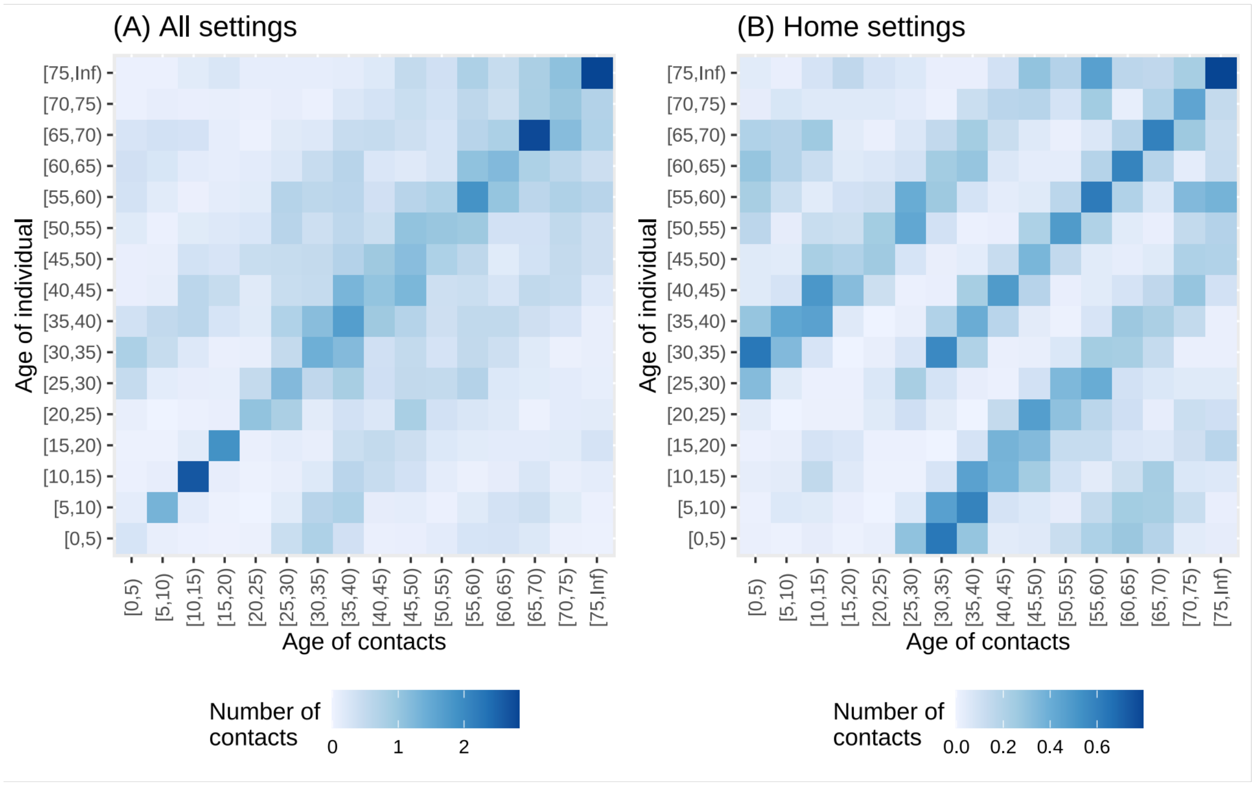

Fig. S4 Smoothed number of contacts between age groups at (A) all settings and (B) only home settings from Zhang et al. (6). They were used to estimate the force of infection.

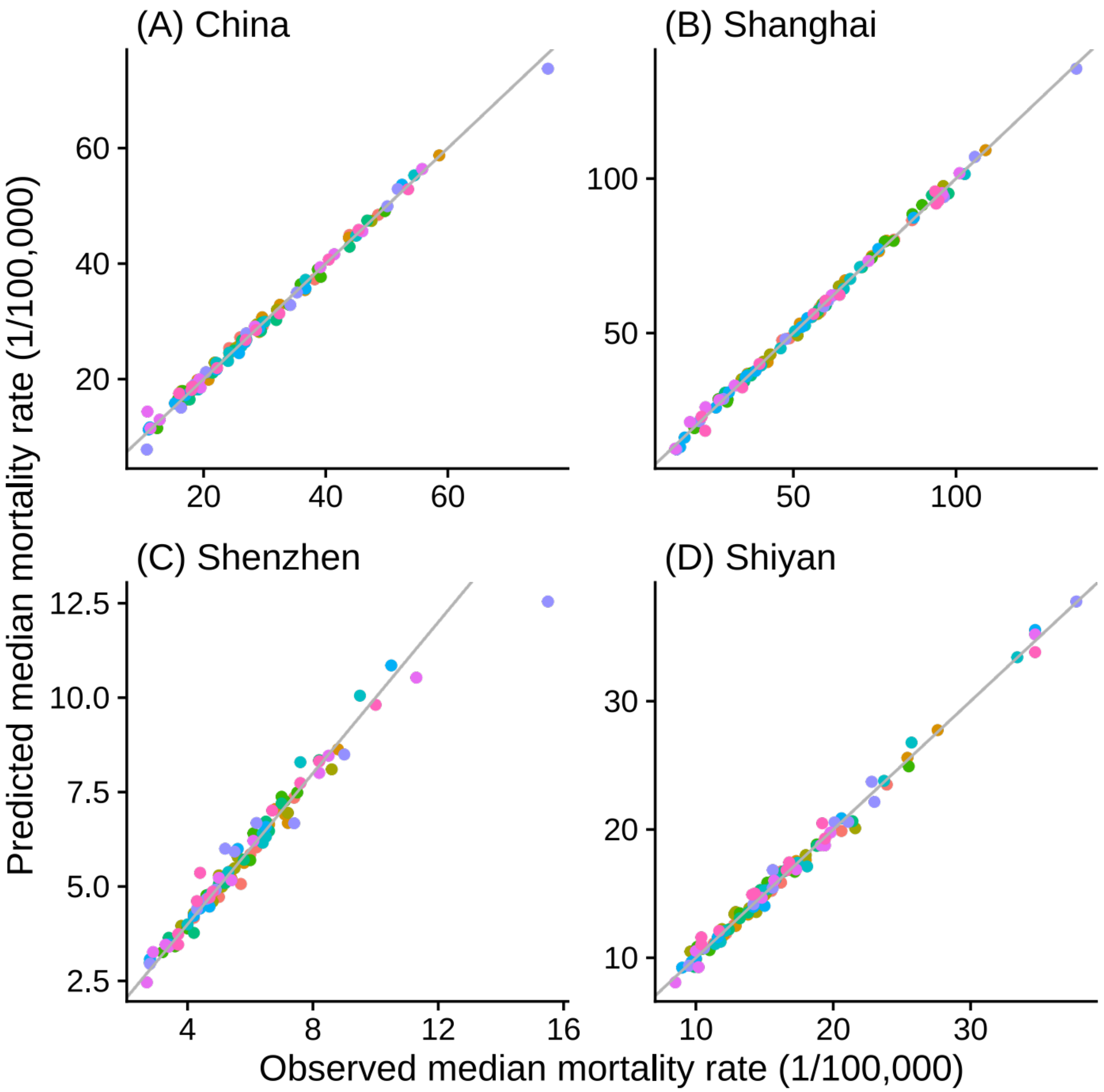

Fig. S5 Median mortality rates by location and sensitivity scenario observed from simulations and predicted by Gaussian process models with samples from the full parameter space under the baseline scenario with a 10-fold cross-validation design. Different colors represent samples from different folds. The gray line in each panel represents the line of Y = X.

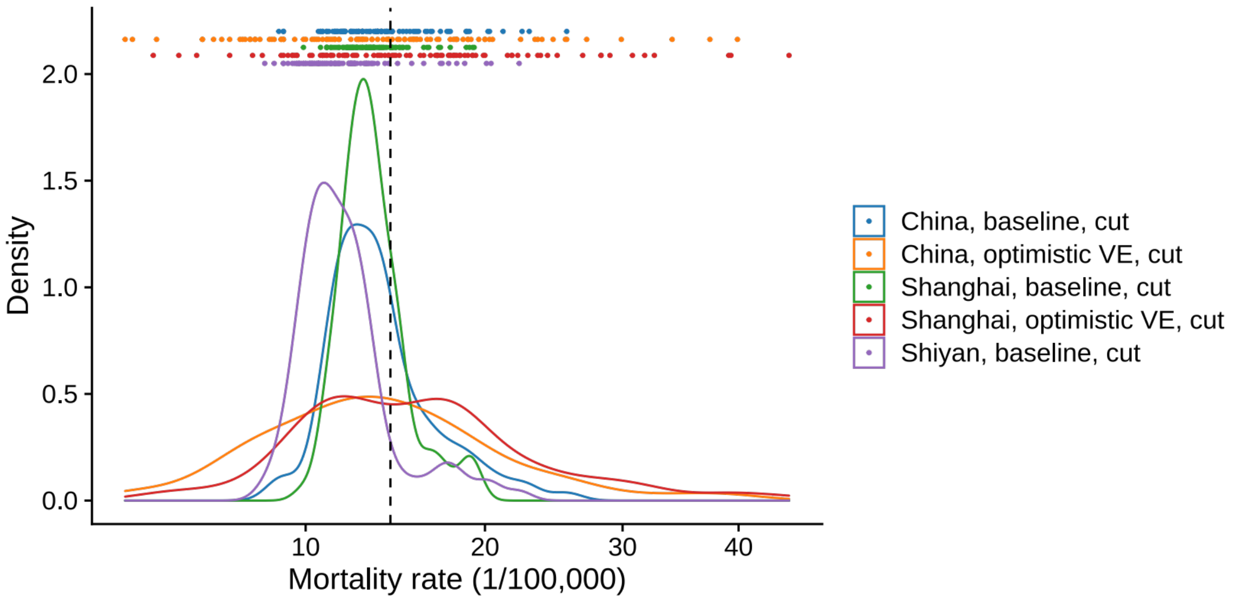

Fig. S6 Median mortality rates (dots on the top of the figure) by location and sensitivity scenarios (colors) for the 100 random samples from the reduced intervention parameter space and their probability density curves (curves). The vertical dashed line represents a mortality rate of 14.3 per 100,000 persons.

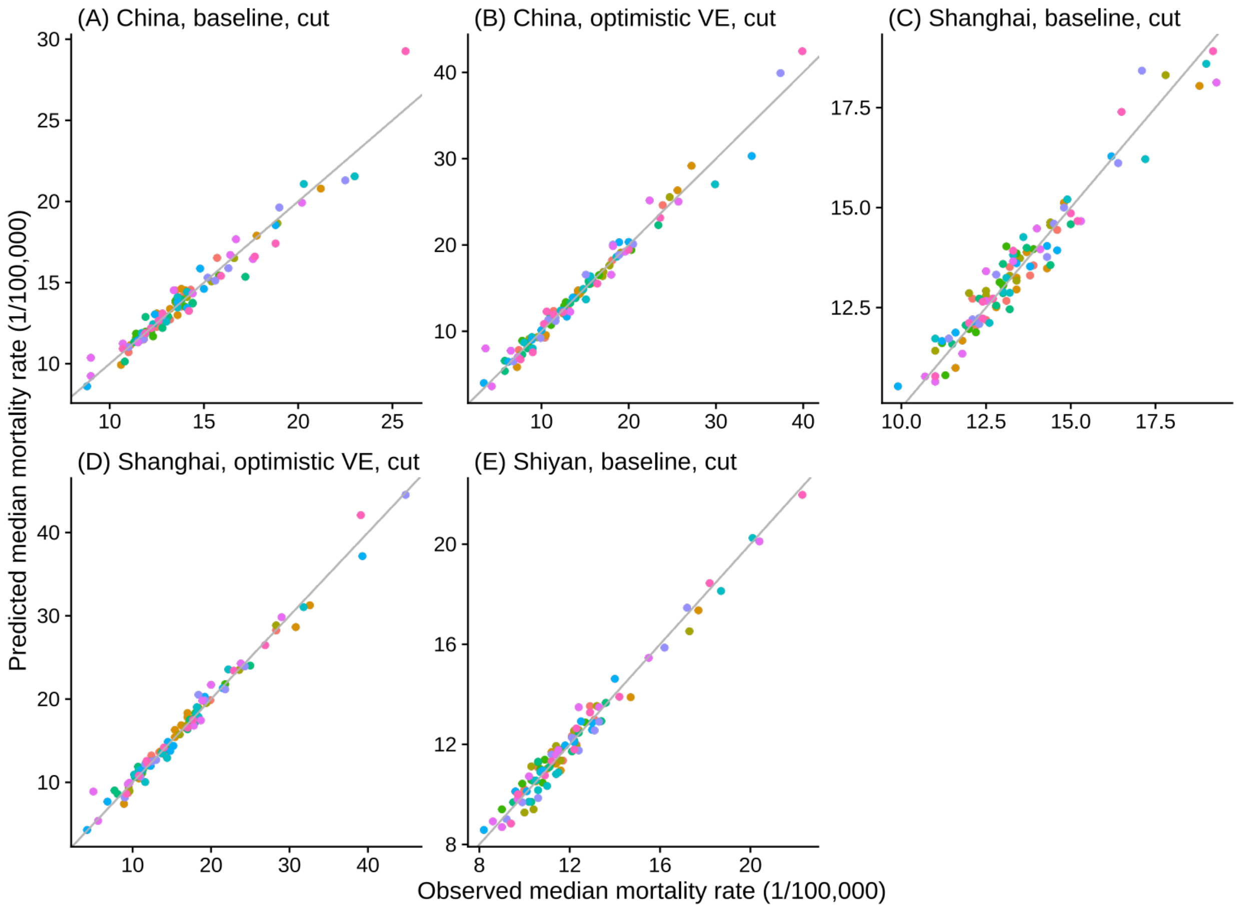

Fig. S7 Median mortality rates by locations and sensitivity scenarios observed from simulations and predicted by Gaussian process models with samples from the reduced parameter space with a 10-fold cross-validation design. Different colors represent samples from different folds. The gray line in each panel represents the line of Y = X.

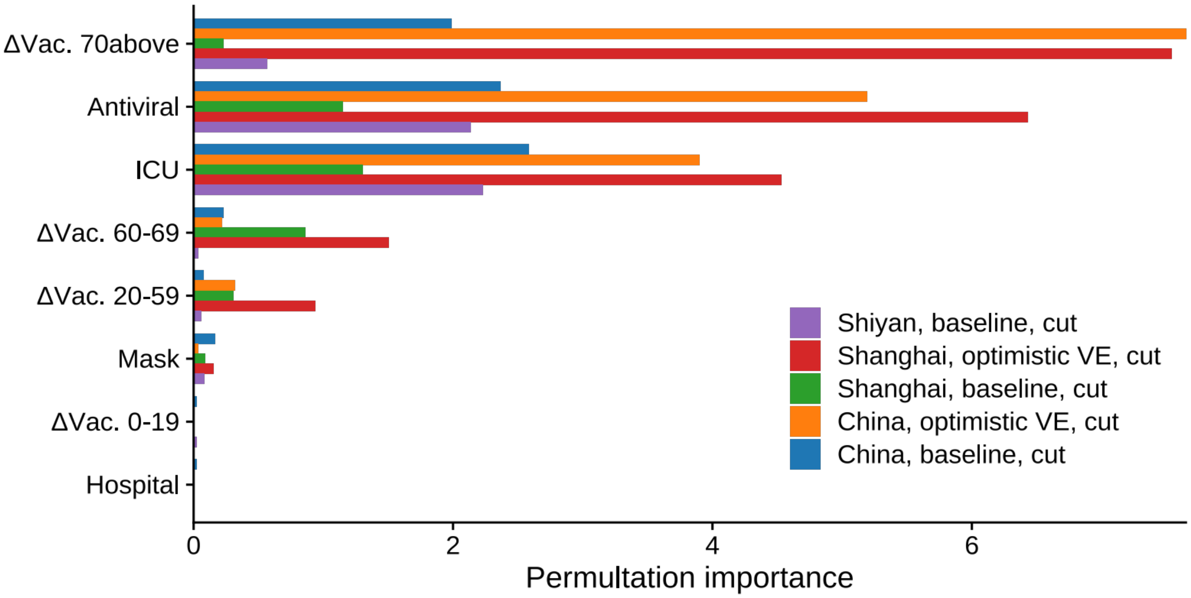

Fig. S8 Permutation importance of each intervention parameter for predicting the median mortality rate (colored bars) in the reduced parameter space. The permutation importance of a parameter represents the amount of increase in the root mean squared prediction error when the randomly shuffled values of this parameter, together with the unchanged values of the other parameters, were used to make the prediction.

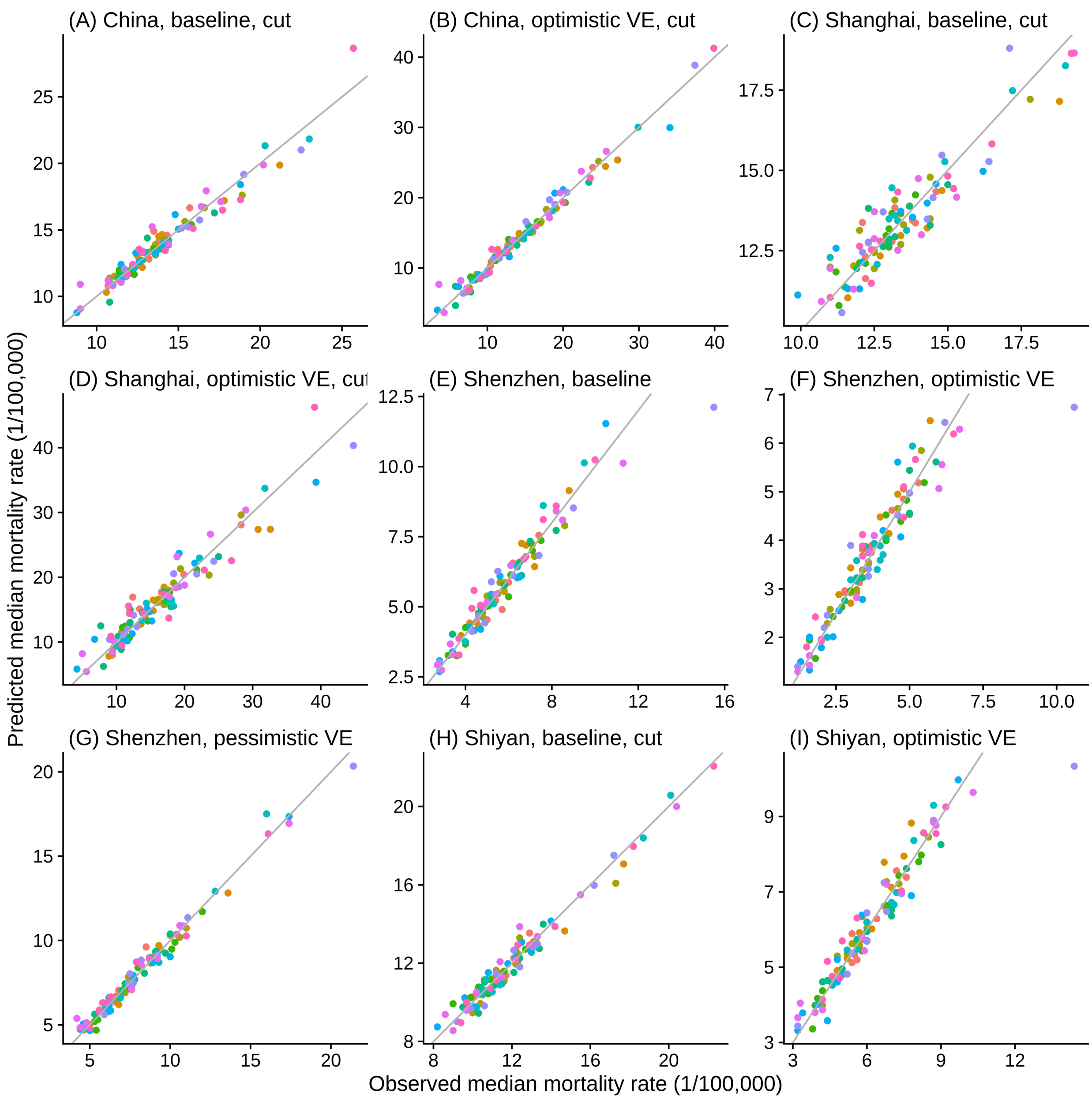

Fig. S9 Median mortality rates by locations and sensitivity scenarios observed from simulations and predicted by Gaussian process models fitted with only the three most important parameters with a 10-fold cross-validation design. Different colors represent samples from different folds. The gray line in each panel represents the line of Y = X.

Table S1. Number of hospital and ICU beds per capita for different locations. The numbers of hospital beds were obtained from China City Statistical Yearbook 2021 (21), while those of ICU beds were obtained from Chen et al. (22).

	Hospital beds per 1,000	ICU beds per 100,000
**China**	5.06	4.37
**Shanghai**	5.78	6.14
**Shenzhen**	3.27	3.42
**Shiyan**	6.82	5.13

Table S2. Parameter values for the sensitivity analysis.

Scenarios	R_0	Rapid antigen test coverage	Vaccine-induced moderator of infection risk (1st, 2nd, 3rd doses)	Vaccine-induced moderator of onward transmission (1st, 2nd, 3rd doses)	Relative susceptibility of children and adolescents when compared with adults (< 10, 10-19 yrs)	Relative infectiousness of asymptomatic cases	Relative contact rate once identified and isolated	Increases in mortality rate when severe cases have no access to hospital beds
**Baseline**	7	100%	0·969, 0·930, 0·869	1, 1, 0·947	1,1	1	0.2	10
**75% testing**	7	75%	0·969, 0·930, 0·869	1, 1, 0·947	1,1	1	0.2	10
**R_0 = 5**	5	100%	0·969, 0·930, 0·869	1, 1, 0·947	1,1	1	0.2	10
**R_0 = 10**	10	100%	0·969, 0·930, 0·869	1, 1, 0·947	1,1	1	0.2	10
**Optimistic VE**	7	100%	0·994, 0·952, 0·908	1, 1, 1	1,1	1	0.2	10
**Pessimistic VE**	7	100%	0·944, 0·909, 0·83	1, 1, 0·894	1,1	1	0.2	10
**Lower child. sus.**	7	100%	0·969, 0·930, 0·869	1, 1, 0·947	0·52, 0·72	1	0.2	10
**Lower asymp. Inf.**	7	100%	0·969, 0·930, 0·869	1, 1, 0·947	1,1	0.3	0.2	10
**No self-isolation**	7	100%	0·969, 0·930, 0·869	1, 1, 0·947	1,1	1	1	10
**5*hosp. mort.**	7	100%	0·969, 0·930, 0·869	1, 1, 0·947	1,1	1	0.2	5
**2*hosp. mort**	7	100%	0·969, 0·930, 0·869	1, 1, 0·947	1,1	1	0.2	2

Table S3. Age-dependent transition rates when not vaccinated. The rates were estimated with the methods in Cai et al. (5), but with the latest age-specific case fatality rates in Hong Kong (23), instead of those in the spring of 2022.

Age	Proportion of asymptomatic cases	Probability of becoming a severe case from a mild case	Probability of becoming a critical case from a mild case	Probability of death from a severe case	Probability of death from a critical case
[0,5)	0.973	0.08	0	0.004	0.034
[5,10)	0.973	0.057	0.001	0.005	0.05
[10,15)	0.973	0.022	0.001	0.007	0.067
[15,20)	0.973	0.022	0.001	0.007	0.1
[20,25)	0.966	0.036	0.002	0.01	0.125
[25,30)	0.966	0.036	0.002	0.01	0.125
[30,35)	0.966	0.046	0.005	0.008	0.104
[35,40)	0.966	0.046	0.005	0.008	0.104
[40,45)	0.954	0.032	0.006	0.032	0.207
[45,50)	0.954	0.032	0.006	0.032	0.207
[50,55)	0.954	0.07	0.017	0.058	0.333
[55,60)	0.954	0.07	0.017	0.058	0.333
[60,65)	0.947	0.079	0.033	0.087	0.364
[65,70)	0.947	0.079	0.033	0.087	0.364
[70,75)	0.947	0.194	0.18	0.25	0.375
[75,Inf)	0.903	0.251	0.234	0.25	0.375

Table S4. Reduction in the transition rates by vaccine dose. The rates were estimated as the mean of the optimistic and pessimistic scenarios from Cai et al.(5)

Dose	Reduction in the proportion of asymptomatic cases			Reduction in the probability of becoming a severe/critical case from a mild case			Reduction in the probability of death from a severe/critical case		
	Baseline	Opt. VE	Pess. VE	Baseline	Opt. VE	Pess. VE	Baseline	Opt. VE	Pess. VE
1	0.065	0.115	0.015	0.392	0.392	0.392	0.153	0.188	0.161
2	0.167	0.196	0.138	0.627	0.710	0.544	0.153	0.208	0.161
3	0.247	0.355	0.138	0.807	0.964	0.650	0.217	0.217	0.217

Table S5. The distribution of waiting times between states from Cai et al. (5) and Kerr et al. (24).

From	To	Distribution (mean, standard deviation)
Exposed	Asymptomatic	Gamma(4, 2.2)
Exposed	Presymptomatic	Gamma(4, 2.2)
Asymptomatic	Recovered	Gamma(6.3, 3.5)
Presymptomatic	Mild	Gamma(1.8, 2)
Mild	Recovered	Gamma(4.5, 2.9)
Mild	Severe	Gamma(2.2, 1)
Mild	Critical	Gamma(2.2, 1)
Severe	Recovered	Gamma(18, 6.3)
Severe	Dead	Gamma(12, 3)
Critical	Recovered	Gamma(18, 6.3)
Critical	Dead	Gamma(10, 4.8)

References

1. Leech G, Rogers-Smith C, Monrad JT, Sandbrink JB, Snodin B, Zinkov R, et al. Mask wearing in community settings reduces SARS-CoV-2 transmission. Proceedings of the National Academy of Sciences. 2022;119(23):e2119266119.
2. Davido B, Dumas L, Rottman M. Modelling the Omicron wave in France in early 2022: Balancing herd immunity with protecting the most vulnerable. Journal of Travel Medicine. 2022;29(3):taac005.
3. Liu Y, Rocklöv J. The effective reproductive number of the Omicron variant of SARS-CoV-2 is several times relative to Delta. Journal of Travel Medicine. 2022;29(3):taac037.
4. Burki TK. Omicron variant and booster COVID-19 vaccines. The Lancet Respiratory Medicine. 2022;10(2):e17.
5. Cai J, Deng X, Yang J, Sun K, Liu H, Chen Z, et al. Modeling transmission of SARS-CoV-2 omicron in China. Nature Medicine. 2022:1-8.
6. Zhang J, Klepac P, Read JM, Rosello A, Wang X, Lai S, et al. Patterns of human social contact and contact with animals in Shanghai, China. Scientific reports. 2019;9(1):1-11.
7. Ye L, Li WF, Shao J, Xu Z, Ju J, Xu H. Fighting Omicron epidemic in China: real-world big data from Fangcang Shelter Hospital during the outbreak in Shanghai 2022. Journal of Infection.
8. Jalali N, Brustad HK, Frigessi A, MacDonald EA, Meijerink H, Feruglio SL, et al. Increased household transmission and immune escape of the SARS-CoV-2 Omicron compared to Delta variants. Nature Communications. 2022;13(1):1-5.

9. Bhatt M, Plint AC, Tang K, Malley R, Huy AP, McGahern C, et al. Household transmission of SARS-CoV-2 from unvaccinated asymptomatic and symptomatic household members with confirmed SARS-CoV-2 infection: an antibody-surveillance study. Canadian Medical Association Open Access Journal. 2022;10(2):E357-E66.
10. Viner RM, Mytton OT, Bonell C, Melendez-Torres G, Ward J, Hudson L, et al. Susceptibility to SARS-CoV-2 infection among children and adolescents compared with adults: a systematic review and meta-analysis. JAMA pediatrics. 2021;175(2):143-56.
11. Sayampanathan AA, Heng CS, Pin PH, Pang J, Leong TY, Lee VJ. Infectivity of asymptomatic versus symptomatic COVID-19. The Lancet. 2021;397(10269):93-4.
12. Nakajo K, Nishiura H. Transmissibility of asymptomatic COVID-19: Data from Japanese clusters. International Journal of Infectious Diseases. 2021;105:236-8.
13. Buitrago-Garcia D, Egli-Gany D, Counotte MJ, Hossmann S, Imeri H, Ipekci AM, et al. Occurrence and transmission potential of asymptomatic and presymptomatic SARS-CoV-2 infections: A living systematic review and meta-analysis. PLoS medicine. 2020;17(9):e1003346.
14. Wang Y, Tian H, Zhang L, Zhang M, Guo D, Wu W, et al. Reduction of secondary transmission of SARS-CoV-2 in households by face mask use, disinfection and social distancing: a cohort study in Beijing, China. BMJ global health. 2020;5(5):e002794.
15. Shao W, Chen X, Zheng C, Liu H, Wang G, Zhang B, et al. Effectiveness of COVID-19 vaccines against SARS-CoV-2 variants of concern in real-world: a literature review and meta-analysis. Emerging Microbes & Infections. 2022;11(1):2383-92.
16. Office of the Leading Group of the State Council for the Seventh National Population Census. Tabulation on 2020 China Population Census by County. Beijing: China Statistics Press; 2022.
17. Shenzhen News. The COVID-19 vaccination coverage in Shenzhen reached two ten millions 2022 [Available from: https://www.sznews.com/news/content/2022-04/29/content_25094112.htm.
18. Shiyan Radio and Television Administration. The booster coverage in Shiyan above 18 years old is over 80 percent 2022 [Available from: http://www.syiptv.com/article/show/180844.
19. The State Council of the People's Republic of China. Press Conference of the Joint Prevention and Control Mechanism of the State Council 2022 [Available from: http://www.gov.cn/xinwen/gwylflkjz200/index.htm.
20. The State Council Information Office of the People's Republic of China. Transcript of the press conference of the Joint Prevention and Control Mechanism of the State Council on December 14, 2022 2022 [Available from: http://www.nhc.gov.cn/xcs/s3574/202212/b927d43bd0354ffeb5a8e1b91377302f.shtml.
21. National Bureau of Statistics of China. China City Satistical Yearbook. Beijing: China Statistics Press; 2021.
22. Chen Y, Li J, Wang X. Predictions of the shortage of healthcare resources in China. Chinese Health Resources. 2021;24(4):453-61.
23. Centre for Health Protection of Hong Kong. Latest situation of COVID-19 (as of 12 January 2023) 2023 [Available from: https://www.chp.gov.hk/files/pdf/local_situation_covid19_en.pdf.
24. Kerr CC, Stuart RM, Mistry D, Abeysuriya RG, Rosenfeld K, Hart GR, et al. Covasim: An agent-based model of COVID-19 dynamics and interventions. PLOS Computational Biology. 2021;17(7):e1009149.
